# Pathogenesis and precision management of osteogenesis imperfecta: from genetic mechanisms to novel therapies

**DOI:** 10.1007/s12519-026-01086-7

**Published:** 2026-09-30

**Authors:** Xue-Qian Wang, Cai Zhang, Xin Li, Yan Sun, Qiong Chen, Bing-Yu Yang, Yao-Shuang Li, Xin-Yi Jiang, Zhong-Hang Zhao, Li Wang, Xiao-Hong Sun, Hong-Ying Wang, Wen-Li Lu, Hai-Yan Wei, Zhe Su, Xiu-Zhi Ren, Bing-Yan Cao, Wei Wu, Ting Chen

**Affiliations:** 1 Suzhou Clinical Center for Rare Diseases in Children, Suzhou, China; 2 Department of Endocrinology, Genetics and Metabolism, Children’s Hospital of Soochow University, Suzhou, China; 3 Department of Pediatrics, Tongji Children’s Hospital, Tongji Hospital, Tongji Medical College, Huazhong University of Science and Technology, Wuhan, China; 4 Hubei Key Laboratory of Pediatric Genetic Metabolic and Endocrine Rare Diseases, Wuhan, China; 5 Hubei Provincial Clinical Research Center for Child Growth, Development, and Metabolic Diseases, Wuhan, China; 6 Department of Endocrinology, Metabolism and Genetics, Shanghai Children’s Medical Center, School of Medicine, Shanghai Jiao Tong University, Shanghai, China; 7 Department of Pediatrics, Shandong Provincial Hospital Affiliated to Shandong First Medical University, Jinan, China; 8 Department of Endocrinology, Genetics and Metabolism, Children’s Hospital Affiliated to Zhengzhou University, Henan Children’s Hospital, Zhengzhou Children’s Hospital, Zhengzhou, China; 9 Department of Endocrinology, Shenzhen Children’s Hospital, Shenzhen, China; 10 Department of Pharmacy, Shenzhen Children’s Hospital, Shenzhen, China; 11 Department of Pediatrics, Ruijin Hospital Affiliated to Shanghai Jiao Tong University, Shanghai, China; 12 Department of Orthopaedics, Children’s Hospital of Soochow University, Suzhou, China; 13 Department of Endocrinology, Children’s Hospital Affiliated to Capital Institute of Pediatrics, Beijing, China; 14 Department of Endocrinology, Children’s Hospital Zhejiang University School of Medicine, Hangzhou, China

**Keywords:** Genotype–phenotype correlation, Molecular pathogenesis, Osteogenesis imperfecta, Precision medicine, Targeted therapies

## Abstract

**Background:**

Osteogenesis imperfecta (OI) is a clinically and genetically heterogeneous skeletal dysplasia characterized by systemic bone fragility. Beyond skeletal manifestations, OI is a multisystem condition involving dentinogenesis imperfecta, hearing loss, joint hyperlaxity, and cardiopulmonary complications. The diagnostic paradigm for OI has undergone a revolutionary shift from traditional clinical phenotyping to a molecular etiology-based framework. Driven by advancements in high-throughput sequencing, mutations in over 20 implicated genes have expanded the current OI nosology to at least 23 distinct types.

**Data sources:**

This narrative review was conducted by searching for papers using PubMed/MEDLINE. Relevant publications were identified using single and/or combined keywords including: osteogenesis imperfecta, genes (such as *COL1A1* and *COL1A2*), animal models, phenotypes (such as osteopenia and femoral bowing), and drug therapies (such as bisphosphonate and denosumab), mesenchymal stem cells, gene therapy, and pluripotent stem cells.

**Results:**

This review systematically delineates the molecular pathogenesis of OI, categorizing defects into disrupted collagen biosynthesis, impaired post-translational modification, aberrant procollagen trafficking, and dysfunctional osteoblast differentiation or mineralization pathways. Of note, we highlight the unique genetic landscape and clinical characterization of OI within Asian populations. Furthermore, we evaluate the transition towards precision management. Surgical management of OI has been optimized by telescopic rodding and a multidisplinary approach. Bisphosphonates remain a cornerstone of medical therapy, though responses vary with OI genotypes. Emerging targeted biologics include denosumab, romosozumab, setrusumab, and fresolimumab. Finally, we discuss the transformative potential of cell-based therapies, particularly mesenchymal stem cells. Precision gene editing technologies, such as CRISPR-Cas9, offer a strategy in addressing the root genetic causes of OI.

**Conclusions:**

Integrating molecular diagnostics with robust genotype–phenotype correlations is essential for advancing personalized care. Future breakthroughs will rely on the translation of gene and cell therapies to ultimately improve the long-term clinical outcomes for patients with OI.

**Graphical abstract:**

**Figure:**
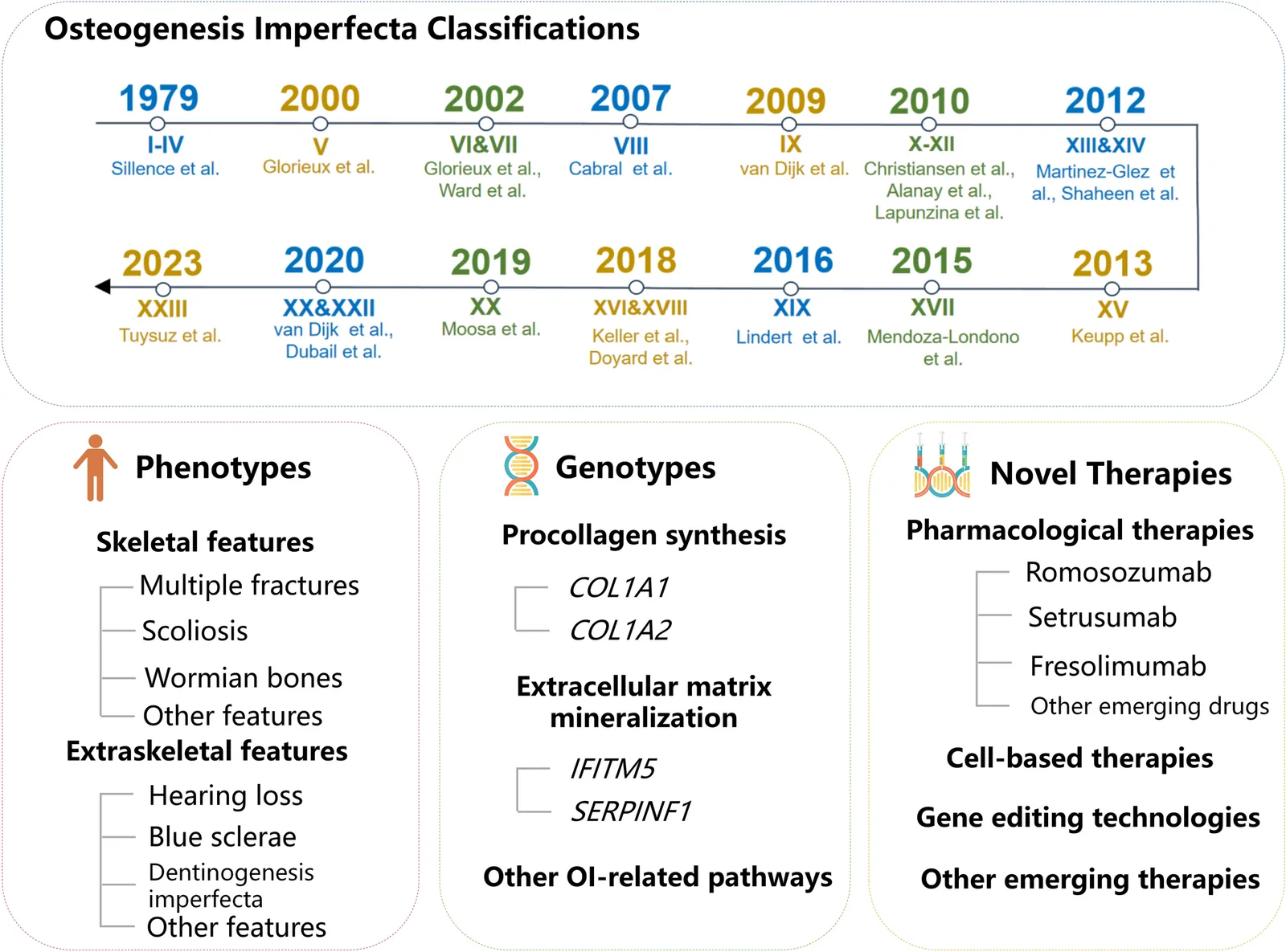

**Supplementary Information:**

The online version contains supplementary material available at https://doi.org/10.1007/s12519-026-01086-7.

## Introduction

Osteogenesis imperfecta (OI) comprises a genetically heterogeneous group of disorders characterized by increased bone fragility and a broad spectrum of associated connective tissue manifestations. The estimated incidence ranges between 1 in 15,000 to 20,000 live births, making it one of the most common skeletal dysplasias associated with fracture predisposition [1]. Historically, OI has been defined by its hallmark clinical feature, susceptibility to fractures of varying severity throughout life, but it is now recognized as a multisystem condition involving dentinogenesis imperfecta (DI), hearing loss, joint hyperlaxity, and cardiopulmonary complications, among others [2].

The classification of OI has undergone a paradigm shift since the landmark Sillence classification in 1979 [1]. While the original types I–IV provided a robust clinical framework, the integration of high-throughput sequencing has revealed an expansive genetic landscape. To date, the broader nosology of genetic skeletal disorders recognizes up to 55 distinct types of OI and related bone fragility syndromes (Supplementary Tables 1 and 2), involving over 30 implicated genes. Indeed, the specific molecular landscape of OI has expanded to include at least 21 genes and 23 distinct clinical types (Table 1). This expanded classification also reflects a paradigm shift in understanding OI pathogenesis. This extends beyond primary collagen synthesis defects to encompass disruptions in procollagen post-translational modification, intracellular trafficking of procollagen, bone mineralization, and osteoblast signaling pathways (Table 2) [3]. Crucially, recent epidemiological data have begun to highlight unique genetic signatures and clinical characteristics within Asian populations, which are essential for refining global diagnostic accuracy.

**Table 1:** Clinical severity and distinct features of osteogenesis imperfecta subtypes

OI type	MIM	First description	Genes	Severity of skeletal involvement	Distinctive features of bone abnormalities	Extra-skeletal manifestations
I	166200	Sillence et al., 1979 [1]	*COL1A1* / *COL1A2*	Mild	Reduced bone density, fractures, mild skeletal deformities	Blue sclerae, hearing loss, dentinogenesis imperfecta, ligamentous laxity
II	166210	Sillence et al., 1979 [1]	*COL1A1* / *COL1A2*	Lethal	Intrauterine fractures, cranial softening, thoracic deformity	Blue sclerae, respiratory failure
III	259420	Sillence et al., 1979 [1]	*COL1A1* / *COL1A2*	Severe	Fractures present at birth, progressive deformities, scoliosis, severe short stature	Blue sclerae, hearing loss, dentinogenesis imperfecta, ligamentous laxity, respiratory complications
IV	166220	Sillence et al., 1979 [1]	*COL1A1* / *COL1A2*	Moderate	Frequent fractures during childhood, skeletal deformities, reduced bone density	Blue sclerae, hearing loss, dentinogenesis imperfecta, ligamentous laxity
V	610967	Glorieux et al., 2000 [4]	*IFITM5*	Mild to severe	Multiple fractures, scoliosis, long-bone deformities, and short stature	Triangular face, bluish sclerae (in some patients), dentinogenesis imperfecta (rare)
VI	613982	Glorieux et al., 2002 [5]	*SERPINF1*	Moderate to severe	Vertebral compression fractures, significant bone deformity, short stature	Bluish sclerae (in some patients), gross motor delay
VII	610682	Ward et al., 2002 [6]	*CRTAP*	Severe to lethal	Intrauterine fractures, extreme osteoporosis, rhizomelic limb shortening	Large open anterior fontanelle, proptosis, bluish sclerae, absent pulmonry artery, hydronephrosis
VIII	610915	Cabral et al., 2007 [7]	*P3H1*	Severe to lethal	Severe growth deficiency, extreme skeletal undermineralization, bulbous metaphyses	White sclerae, round face, barrel-shaped chest
IX	259440	Van Dijk et al., 2009 [8]	*PPIB*	Severe to lethal	Multiple fractures, bowing of long bones, osteopenia	Short limb dwarfism, white to gray sclerae
X	613848	Christiansen et al., 2010 [9]	*SERPINH1*	Severe to lethal	Multiple fractures at birth, severe osteopenia, limb bowing, dentinogenesis imperfecta	Blue sclerae, dentinogenesis imperfecta, renal complications, chronic lung disease
XI	610968	Alanay et al., 2010 [10]	*FKBP10*	Moderate to severe	Joint contractures, dentinogenesis imperfecta	Triangular face, white to faintly blue sclera
XII	613849	Lapunzina et al., 2010 [11]	*SP7*	Mild to moderate	Recurrent fractures, mild bone deformations, generalized osteoporosis	Delayed teeth eruption, progressive hearing loss
XIII	614856	Martinez-Glez et al., 2012 [12]	*BMP1*	Moderate to severe	Severe growth deficiency, borderline osteoporosis, multiple fractures, severe bone deformity	Delayed gross motor development (in some patients)
XIV	615066	Shaheen et al., 2012 [13]	*TMEM38B*	Mild to moderate	Multiple fractures, osteopenia	Blue-grey sclerae, mild (in some patients), muscle wasting, trianglular face
XV	615220	Keupp et al., 2013 [14]	*WNT1*	Moderate to severe	Increased bone fragility, progressive skeletal deformities, short stature, and dentinogenesis imperfecta	Ptosis, brainstem-cerebellar hypoplasia
XVI	616229	Keller et al., 2018 [15]	*CREB3L1*	Severe to lethal	Multiple fractures, decreased ossification of the skull, scoliosis	Blue sclerae, joint hyperlaxity
XVII	616507	Mendoza-Londono et al., 2015 [16]	*SPARC*	Moderate	Spinal compression fracture, scoliosis, short stature	Muscle weakness, hypotonia, and delayed motor development, soft skin, joint hyperlaxity
XVIII	617952	Doyard et al., 2018 [17]	*TENT5A*	Severe to lethal	Congenital bowing of the long bones, wormian bones, vertebral collapse, multiple fractures in the first years of life	Blue sclerae, joint hyperlaxity, large umbilical hernia
XIX	301014	Lindert et al., 2016 [18]	*MBTPS2*	Severe	Prenatal fractures, generalized osteopenia, short stature, scoliosis, thoracic deformity	Blue sclerae (uncommon)
XX	618644	Moosa et al., 2019 [19]	*MESD*	Severe to lethal	Osteopenia, frequent fractures, over tubulation, scoliosis, narrowed thorax	Pulmonary hypoplasia, blue sclerae in infancy, triangular face, retrognathia
XXI	619131	Van Dijk et al., 2020 [20]	*KDELR2*	Moderate	Multiple fractures, disproportionate short stature, scoliosis	Neurodevelopmental delay
XXII	619795	Dubail et al., 2020 [21]	*CCDC134*	Severe	Intrauterine growth retardation, short stature, multiple fractures	Intrauterine growth retardation, sensorineural hearing loss, white–gray-blue sclerae, ovarian inguinal hernia
XXIII	620639	Tuysuz et al., 2023 [22]	*PHLDB1*	Mild	Osteopenia, short and bowed long bones, widened metaphyses	Truncal obesity, blue sclerae (in infancy), acanthosis nigricans, motor delay

*OI* osteogenesis imperfecta, *MIM* Mendelian Inheritance in Man number, *COL1A1* collagen type I alpha 1 chain, *COL1A2* collagen type I alpha 2 chain, *IFITM5* interferon-induced transmembrane protein 5, *SERPINF1* serpin peptidase inhibitor, clade F, member 1, *CRTAP* cartilage-associated protein, *P3H1* prolyl 3-hydroxylase 1, *PPIB* peptidyl-prolyl isomerase B, *SERPINH1* serpin peptidase inhibitor, clade H, member 1, *FKBP10* FK506-binding protein 10, *SP7* specificity protein 7, *BMP1* bone morphogenetic protein 1, *TMEM38B* transmembrane protein 38B, *WNT1* wingless-type MMTV integration site family, member 1, *CREB3L1* cAMP-responsive element-binding protein 3-like 1, *SPARC* secreted protein acidic and cysteine rich, *TENT5A* terminal nucleotidyltransferase 5A, *MBTPS2* membrane-bound transcription factor peptidase, site 2, *MESD* mesoderm development LRP chaperone, *KDELR2* KDEL endoplasmic reticulum protein retention receptor 2, *CCDC134* coiled-coil domain-containing protein 134, *PHLDB1* pleckstrin homology-like domain, family B, member 1

**Table 2:** Genetic classification of osteogenesis imperfecta and underlying pathogenesis

OI type	Genes	MIM	Molecular basis elucidated (year)	Chromosome location	Inheritance pattern	Pathogenesis	Animal model
I	*COL1A1* / *COL1A2*	120150/120160	Byers et al., 1993 [59]	17q21.33/7q21.3	AD	Procollagen synthesis	*Col1a2*^*tm1.1Mcbr*^ mouse model [23]
II	*COL1A1* / *COL1A2*	120150/120160	Chu et al., 1983 [24]	17q21.33/7q21.3	AD	Procollagen synthesis	*Tg(Col1a1-G859C)* and *Tg(Col1a1-G859R)* mouse models [25]
III	*COL1A1* / *COL1A2*	120150/120160	Starman et al., 1989 [26]	17q21.33/7q21.3	AD	Procollagen synthesis	*Col1a1*^*Aga2*^ mouse model [27]
IV	*COL1A1* / *COL1A2*	120150/120160	Wenstrup et al., 1986 [28]	17q21.33/7q21.3	AD	Procollagen synthesis	*Col1a1*^*BrtIIV/*+^ mouse model [29]
V	*IFITM5*	614757	Cho et al., 2012 [30]	11p15.5	AD	Extracellular matrix mineralization	Conditional Rosa26 knockin *mIfitm5* mouse model mouse model mouse model [31], and an inducible *Ifitm5* (c.-14C > T) mouse model[32]
VI	*SERPINF1*	172860	Rauch et al., 2012 [33]	17p13.3	AR	Extracellular matrix mineralization	PEDF^−/−^ mouse model [34, 35]
VII	*CRTAP*	605497	Morello et al., 2006 [36]	3p22.3	AR	Procollagen post-translational modification	*Crtap*^*−/−*^ mouse model [36, 37]
VIII	*P3H1*	610339	Cabral et al., 2007 [7]	1p34.2	AR	Procollagen post-translational modification	*P3h1*^*−/−*^ mouse model [38, 39]
IX	*PPIB*	123841	Van Dijk et al., 2009 [8]	15q22.31	AR	Procollagen post-translational modification	*Ppib*^*−/−*^ mouse model [40, 41]
X	*SERPINH1*	600943	Christiansen et al., 2010 [9]	11q13.5	AR	Procollagen processing and crosslinking	*Hsp47* conditional knockout mouse model: *Hsp47*^*flox/flox*^*; Col2a1-Cre* [42]
XI	*FKBP10*	607063	Alanay et al., 2010 [23]	17q21.2	AR	Procollagen processing and crosslinking	*Fkbp10* conditional knockout mouse: *Scx-Cre; Fkbp10*^*f/f*^ [43]
XII	*SP7*	606633	Lapunzina et al., 2010 [11]	12q13.13	AR	Osteoblast differentiation and function	*Osx*^*−/−*^ mouse model [44]
XIII	*BMP1*	112264	Martinez-Glez et al., 2012 [12]	8p21.3	AR	Procollagen processing and crosslinking	*Bmp1* and *Tll1* conditional knockout mouse model: *Bmp1*^*flox/flox*^*; Tll1*^*flox/flox*^*; UBC-Cre*^*ERT2*^ [45]
XIV	*TMEM38B*	611236	Volodarsky et al., 2013 [46]	9q31.2	AR	Osteoblast differentiation and function	*Tric-b*^*−/−*^ mouse model [47]
XV	*WNT1*	164820	Pyott et al., 2013 [48]	12q13.12	AR	Wnt/β-catenin signaling pathway	*Wnt1*^*sw/sw*^ mouse model [49]
XVI	*CREB3L1*	616215	Keller et al., 2018 [15]	11p11.2	AR	Osteoblast differentiation and function	*Oasis*^*−/−*^ mouse model [50]
XVII	*SPARC*	182120	Mendoza-Londono et al., 2015 [16]	5q33.1	AR	Osteoblast differentiation and function	*Sparc*^*−/−*^ mouse model [51]
XVIII	*TENT5A*	611357	Doyard et al., 2018 [17]	6q14.1	AR	Induction of target gene transcription and bone formation	*Tent5a*^*−/−*^ mouse model [52]
XIX	*MBTPS2*	300294	Lindert et al., 2016 [18]	Xp22.12	XLR	Collagen secretion and post-translational modification	*Mbtps2*^+*/455S *^and *Mbtps2*^+*/ko*^ mouse models [53]
XX	*MESD*	607783	Moosa et al., 2019 [19]	15q25.1	AR	Wnt signaling pathway	*Mesd*^*−/−*^ mouse model, embryonic lethality [54]
XXI	*KDELR2*	609024	Van Dijk et al., 2020 [20]	7p22.1	AR	Collagen folding and intracellular trafficking	No mouse model
XXII	*CCDC134*	618788	Dubail et al., 2020 [21]	22q13.2	AR	RAS/MAPK signaling pathway and collagen synthesis	*Ccdc134*^*−/−*^ mouse model, embryonic lethality [55]
XXIII	*PHLDB1*	612834	Tuysuz et al., 2023 [22]	11q23.3	AR	Collagen synthesis and osteogenic differentiation	No mouse model

*OI* osteogenesis imperfecta, *MIM* Mendelian Inheritance in Man number, *COL1A1* collagen type I alpha 1 chain, *COL1A2* collagen type I alpha 2 chain, *IFITM5* interferon-induced transmembrane protein 5, *SERPINF1* serpin peptidase inhibitor, clade F, member 1, *CRTAP* cartilage-associated protein, *P3H1* prolyl 3-hydroxylase 1, *PPIB* peptidyl-prolyl isomerase B, *SERPINH1* serpin peptidase inhibitor, clade H, member 1, *FKBP10* FK506-binding protein 10, *SP7* specificity protein 7, *BMP1* bone morphogenetic protein 1, *TMEM38B* transmembrane protein 38B, *WNT1* wingless-type MMTV integration site family, member 1, *CREB3L1* cAMP response element-binding protein 3-like 1, *SPARC* secreted protein, acidic, cysteine-rich, *TENT5A* terminal nucleotidyltransferase 5A, *MBTPS2* membrane-bound transcription factor protease, site 2, *MESD* mesoderm development LRP chaperone, *KDELR2* KDEL endoplasmic reticulum protein retention receptor 2, *CCDC134* coiled-coil domain-containing protein 134, *PHLDB1* pleckstrin homology-like domain, family B, member 1, *AD* autosomal dominant, *AR* autosomal recessive, *XLR* X-linked recessive, *Tg* transgenic, *PEDF* pigment epithelium-derived factor, *Osx* Osterix (alternative symbol of SP7), *Tll1* tolloid-like 1, *Cre* causes recombination, *UBC* ubiquitin C, *ERT2* estrogen receptor mutant 2, *Tric-b* trimeric intracellular cation B, *sw* swaying, *OASIS* old astrocyte specifically induced substance (an alternative symbol of CREB3L1).

A precise molecular diagnosis of OI is no longer a luxury but a cornerstone of precision management. Genotype–phenotype correlations now dictate individualized treatment trajectories: for instance, while bisphosphonates remain the mainstay for classical OI, their efficacy varies significantly across different genetic subtypes. This has catalyzed the development of targeted pharmacotherapies, including receptor activator of nuclear factor-κB ligand (RANKL) inhibitors, sclerostin inhibitors, and transforming growth factor-β (TGF-β) inhibitor [56, 57]. Furthermore, modern management advocates for a synergistic approach combining optimized orthopedic interventions with emerging biotechnologies, such as precision gene editing and cell-based therapies, to address the root genetic defects.

In summary, the management of OI has transitioned from clinical empiricism to a framework of molecularly driven precision medicine. This review systematically describes the evolving genetic landscape of OI, examines specific clinical manifestations within Asian cohorts, and evaluates how these molecular insights are transforming the diagnostic, therapeutic, and counseling standards in clinical practice.

## Expanding genetic landscape of osteogenesis imperfecta: from collagen synthesis to cellular signaling

### Molecular pathogenesis and clinical correlation with *COL1A1* and *COL1A2* mutations

**Fig. 1:**
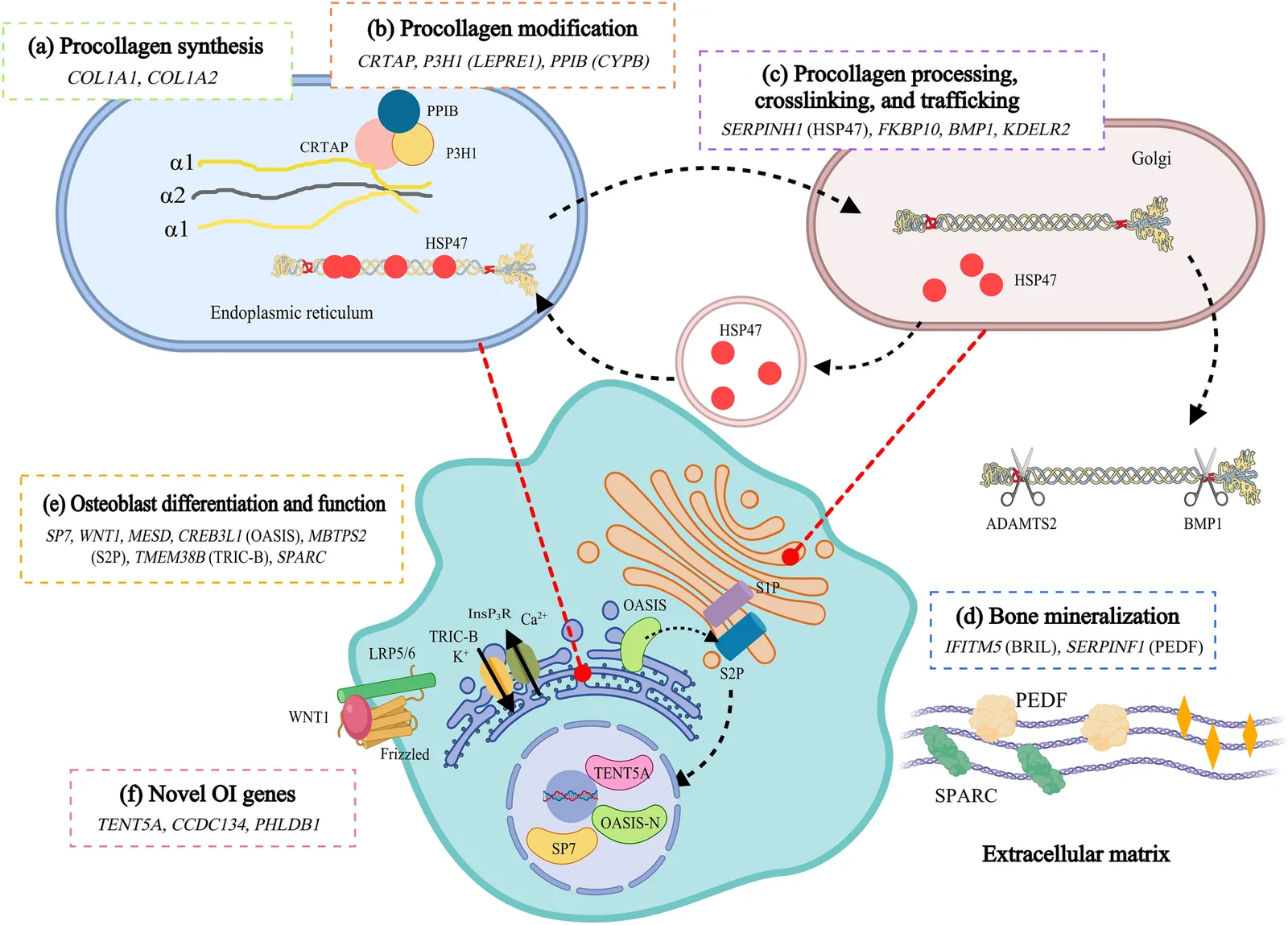
Schematic diagram of genes involved in osteogenesis imperfecta. **a** Type I procollagen synthesis: type I procollagen consists of two pro-α1 chains and one pro-α2 chain; **b** type I procollagen modification: after translation, pro-α1 and pro-α2 chains are processed in the rER. P3H1, CRTAP, and PPIB form a trimeric complex that hydroxylates specific proline residues at the 3-carbon position; **c** procollagen processing, crosslinking, and trafficking: HSP47 binds to the triple-helical domain in the endoplasmic reticulum, preventing aggregation and facilitating transport to the Golgi apparatus. FK506-binding protein 65, a peptidyl-prolyl cis–trans isomerase, modulates LH2 activity, which hydroxylates lysine residues in the N- and C-telopeptides—essential for subsequent cross-linking in the extracellular matrix. After secretion, procollagen is incorporated into the extracellular matrix. The N-propeptide is cleaved by ADAMTS-2, and the C-propeptide by BMP1, resulting in mature type I collagen monomers that undergo fibril assembly and cross-linking. The KDEL receptor 2 is essential for the retrograde transport of ER-resident proteins from the Golgi apparatus back to the ER; **d** bone mineralization: PEDF, a secreted anti-angiogenic factor, and BRIL, a key regulator of mineralization, collaboratively maintain bone homeostasis; **e** osteoblast differentiation and function: secreted WNT1 binds to the Frizzled and LRP5/6 receptor complex on osteoblast precursors, triggering transcriptional programs that drive osteoblast differentiation. This process is supported by MESD, an ER-resident chaperone essential for the correct folding and trafficking of the Wnt co-receptors LRP5/6. Under ER stress, the transcription factor OASIS is translocated to the Golgi apparatus, where Endopeptidase S2P mediates RIP. The liberated N-terminal fragment of OASIS subsequently activates downstream osteogenic genes. SP7—a zinc-finger transcription factor acting downstream of RUNX2 that is indispensable for functional osteoblast differentiation. ER calcium flux is regulated by TRIC-B (a monovalent cation channel) and the InsP3R receptor. SPARC acts as a multifunctional collagen chaperone and ECM glycoprotein, governing procollagen processing, fibrillogenesis, and the maintenance of balanced bone remodeling; **f** emerging novel OI genes with uncertain pathogenicity: TENT5A is a cytoplasmic terminal adenylyltransferase that maintains bone-related mRNA stability via polyadenylation. CCDC134 is a secreted regulator of osteoblast differentiation; loss disrupts the RAS/MAPK pathway. PHLDB1 is a scaffold protein with a C-terminal PH domain that binds PtdIns(3,4,5)P_3_ for membrane recruitment. *rER* rough endoplasmic reticulum, *P3H1* prolyl 3-hydroxylase, *LEPRE1* leucine-and proline-enriched proteoglycan 1 (an alternative symbol of *P3H1*), *CRTAP* cartilage-associated protein, *PPIB* peptidyl-prolyl isomerase B, *CYPB* cyclophilin B (an alternative symbol of *PPIB*), *SERPINH1* serpin peptidase inhibitor, clade H, member 1, *FKBP10* FK506-binding protein 10, *HSP47* heat shock protein 47 (protein symbol of *SERPINH1*), *LH2* lysyl hydroxylase 2, *ADAMTS-2* a disintegrin-like and metalloproteinase with thrombospondin type 1 motif 2, *BMP1* bone morphogenetic protein 1, *KDELR2* KDEL endoplasmic reticulum protein retention receptor 2, *IFITM5* interferon-induced transmembrane protein 5, *SERPINF1* serpin peptidase inhibitor, clade f, member 1, *PEDF* pigment epithelium-derived factor (protein symbol of *SERPINF1*), *BRIL* bone-restricted interferon-induced transmembrane protein-like protein (protein symbol of *IFITM5*), *WNT1* wingless-type MMTV integration site family, member 1, *LRP5/6* low density lipoprotein receptor-related protein 5, *CREB3L1* cAMP response element-binding protein 3-like 1, *OASIS* old astrocyte specifically induced substance (an alternative symbol of *CREB3L1*), *MBTPS2* membrane-bound transcription factor protease, site 2*, S2P* Site-2 protease (protein symbol of *MBTPS2*), *RIP* regulated intramembrane proteolysis, *SP7* specificity protein 7, *RUNX2* runt-related transcription factor 2, *TMEM38B* transmembrane protein 38B, *TRIC-B* trimeric intracellular cation B (protein symbol of *TMEM38B*), *SPARC* secreted protein, acidic, cysteine-rich, *ECM* extracellular matrix, *TENT5A* terminal nucleotidyltransferase 5A, *KDELR2* KDEL endoplasmic reticulum protein retention receptor 2, *CCDC134* coiled-coil domain-containing protein 134, *MAPK* mitogen-activated protein kinase, *PHLDB1* pleckstrin homology-like domain, family B, member 1. This figure was created with MedPeer (medpeer.cn)

OI is predominantly caused by heterozygous dominant mutations in the collagen type I alpha 1 chain (*COL1A1*) [Mendelian Inheritance in Man (MIM) 120150] and collagen type I alpha 2 chain (*COL1A2*) (MIM 120160) procollagen genes, accounting for 80%–85% of cases and primarily manifesting as Sillence types I through IV (MIM 166200, 166210, 259420, 166220). These genes encode the pro-α1(I) and pro-α2(I) chains, which assemble into the triple-helical type I procollagen. *COL1A1* (17q21.33) and *COL1A2* (7q21.3) were fully cloned in the late 1980s, providing the foundation for understanding their genomic architecture [58] (Fig. 1a). Pathogenic variants are dichotomized into quantitative defects (haploinsufficiency) and qualitative defects (dominant-negative). In haploinsufficiency, typically caused by nonsense or frameshift mutations, nonsense-mediated mRNA decay reduces the synthesis of structurally normal collagen by half. Conversely, qualitative defects involve missense substitutions, most commonly replacing a critical glycine with a bulkier amino acid within the Gly-X-Y repeats, which severely disrupt the folding of the triple helix [60].

The pathophysiology of qualitative mutations extends beyond simple matrix deficiency. Misfolded procollagen molecules undergo prolonged enzymatic over-modification (hyperhydroxylation and hyperglycosylation) and are retained within the endoplasmic reticulum (ER) [61]. This intracellular accumulation triggers chronic ER stress, activating the unfolded protein response (UPR) and autophagy pathways. Persistent UPR activation, marked by the upregulation of chaperones like binding immunoglobulin protein and signaling through the protein kinase R-like endoplasmic reticulum kinase (PERK) or inositol-requiring transmembrane kinase/endoribonuclease 1α (IRE1α) pathways, induces osteoblast dysfunction and apoptosis [62]. Furthermore, the over-modified collagen that is secreted into the extracellular matrix (ECM) forms disorganized, "twirled" fibrils that fail to undergo proper mineralization, leading to increased bone fragility and a state of high-turnover [62].

Clinically, the hallmark of OI is lifelong fracture susceptibility, accompanied by variable features including blue sclerae, hearing loss, DI, and skeletal deformities [63]. Clinical severity is fundamentally dictated by mutation type: haploinsufficiency typically results in the mildest form, type I, characterized by non-deforming fractures and near-normal stature. In contrast, qualitative defects generally lead to more severe phenotypes (types II–IV). Type II is perinatally lethal, marked by crumpled long bones and deficient ossification. Type III is a severe, progressively deforming form with "popcorn" metaphyses and significant growth failure. Type IV presents with moderate severity and variable deformities [1, 64].

Of note, rare variants in the C-propeptide cleavage site (e.g., *COL1A1* p.Asp1219Asn) present a distinct "high bone mass" phenotype. These mutations prevent bone morphogenetic protein 1 (BMP1)-mediated removal of the C-propeptide, which is essential for normal fibrillogenesis. The resulting aberrant matrix paradoxically traps minerals, leading to increased bone mineral density (BMD) despite the high fracture risk. These patients often lack the classic extra-skeletal hallmarks of OI, such as blue sclerae or DI, posing a diagnostic challenge [65].

### Disruptions in the bone mineralization pathway

#### Osteogenesis imperfecta type V (*IFITM5*)

OI type V (MIM 610967) is a distinct autosomal dominant bone fragility disorder primarily caused by a recurrent heterozygous mutation (c.-14C > T) in the 5' untranslated region (UTR) of the interferon-induced transmembrane protein 5 (*IFITM5*) gene (MIM 614757) [30, 66] (Fig. 1d). This specific transition creates an alternative translation initiation codon, resulting in the addition of a novel five-amino-acid sequence (MALEP) to the N-terminus of the bone-restricted interferon-induced transmembrane protein-like protein (BRIL). This MALEP-BRIL variant exerts a neomorphic function that fundamentally corrupts skeletal progenitor fate [67]. Recent mechanistic insights from sophisticated conditional mouse models have shown that the expression of mutant BRIL in osteochondral progenitors triggers aberrant extracellular signal-regulated kinase (ERK)–SRY-related high-mobility group box 9 (SOX9) signaling, leading to cartilage overgrowth and joint deformities [31]. Crucially, a 2025 study by Tan et al. utilizing single-cell RNA sequencing identified a unique hybrid cell population, "chondrocyte-like osteoblasts", which co-express osteogenic and chondrogenic markers, confirming that the mutation drives ectopic chondrogenesis at the expense of normal bone formation and shifts mesenchymal differentiation toward adipogenesis [32].

**Fig. 2:**
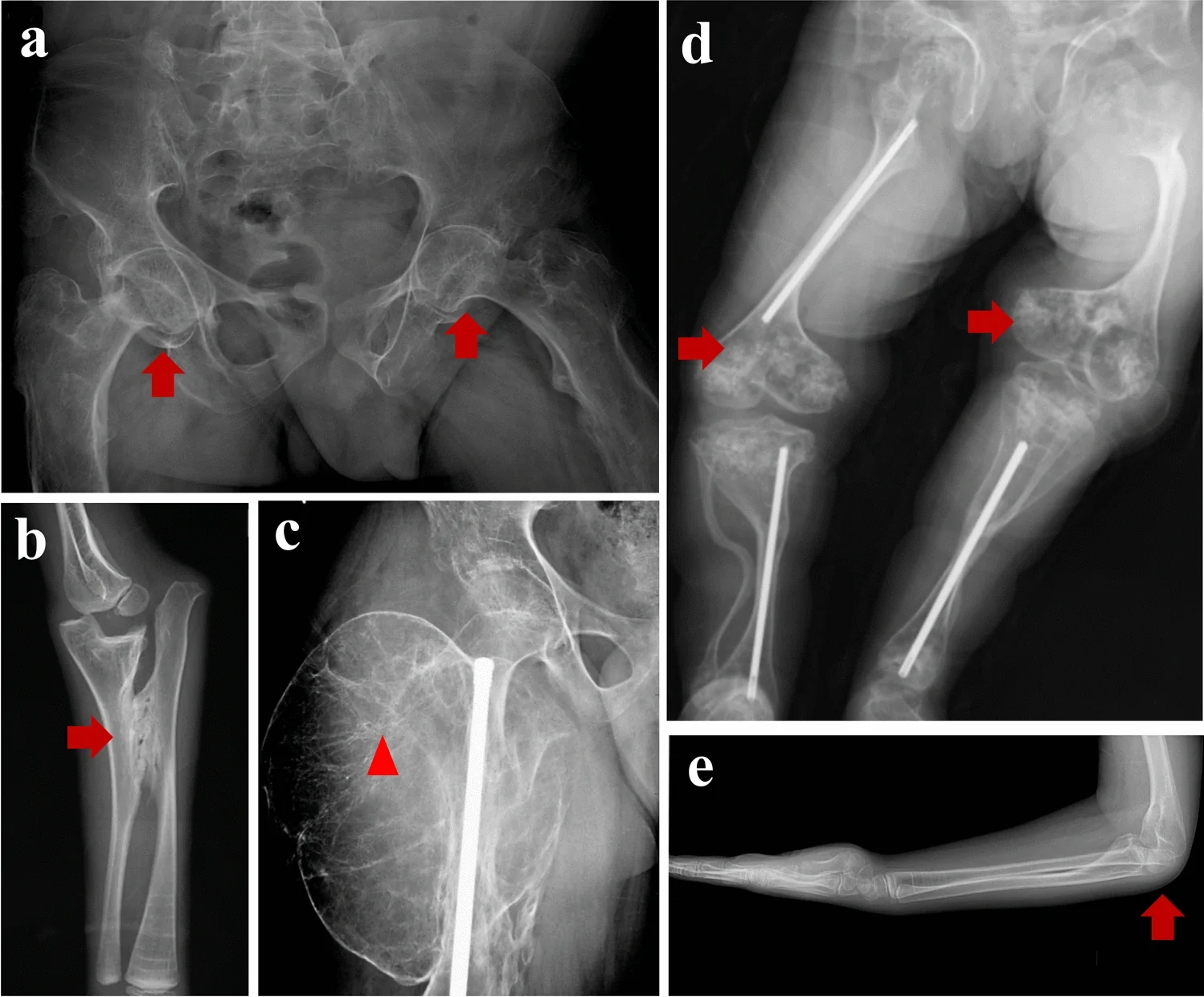
Characteristic radiographic manifestations of a patient with osteogenesis imperfecta type V. **a** Pelvic radiograph with hip joint fusion (red arrows); **b** forearm radiograph revealing interosseous membrane calcification (red arrow); **c** radiograph showing massive callus formation (red triangle); **d** lower extremity radiographs illustrating "popcorn-like" calcifications in the metaphyses (red arrows); **e** forearm radiograph depicting radial head dislocation (red arrow)

Clinically, OI type V is characterized by a unique triad of radiological signs: post fracture hypertrophic callus (HPC) formation, calcification of the interosseous membranes (particularly in the forearm), and bilateral radial head dislocation, which leads to restricted forearm rotation [67, 68]. Unlike *COL1A1-* or *COL1A2*-related OI, patients typically lack blue sclerae and dentinogenesis imperfecta but suffer from moderate to severe bone fragility, scoliosis, and short stature (Fig. 2). Despite the uniformity of the recurrent c.-14C > T mutation, OI type V exhibits pronounced phenotypic heterogeneity both between and within families. For instance, while bone mass is generally reduced, height *Z*-scores and ambulation status range from near-normal to severely compromised. In addition, the prevalence of classic hallmarks like HPC is inconsistent, appearing in only approximately 65% of cases [67]. This symptomatic variability likely arises from a complex interplay of genetic modifiers, such as interactions with *FKBP11*, as well as epigenetic factors and gender-specific differences, with males often exhibiting lower BMD and more severe growth impairment [69, 70]. Furthermore, individual inflammatory responses during fracture healing and the timing of bisphosphonate interventions may further contribute to the diverse clinical outcomes observed in this specific genotype [70].

#### Osteogenesis imperfecta type VI (*SERPINF1*)

**Fig. 3:**
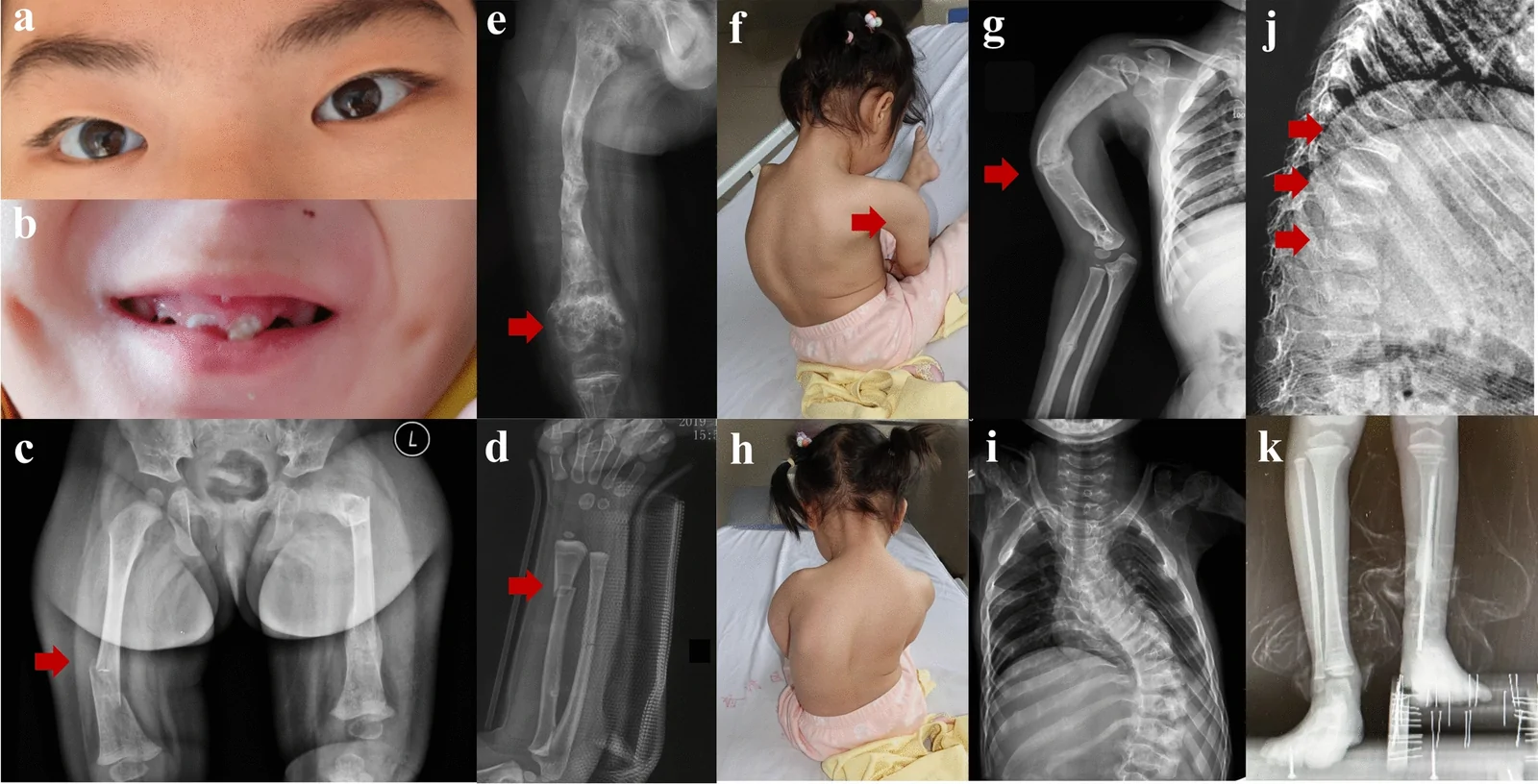
Clinical features of patients with osteogenesis imperfecta type VI. **a** Light blue sclerae; **b** dentinogenesis imperfecta; **c**, **d** radiographic findings of long bone fractures (red arrows); **e** "popcorn-like" metaphyseal changes in the femoral metaphysis, a distinctive radiological hallmark of abnormal endochondral ossification (red arrow); **f**, **g** humeral curvature deformity with concurrent fracture (red arrows); **h**, **i** scoliosis associated with rib cage "ribboning" (thin, abnormally shaped ribs); **j** biconcave vertebral compression fractures (red arrows); **k** lower limb length discrepancy

OI type VI (MIM 613982) is one of the most prevalent autosomal recessive forms of the disease, caused by biallelic loss-of-function mutations in the serpin peptidase inhibitor, clade F, member 1 (*SERPINF1*) gene (MIM 172860) on chromosome 17p13.3 [34, 71–73]. This gene encodes pigment epithelium-derived factor (PEDF), a secreted glycoprotein that maintains bone homeostasis by inhibiting sclerostin expression and modulating osteoid mineralization [33, 74]. Most causative mutations are null, resulting in undetectable serum PEDF levels, which serves as a specific and sensitive diagnostic biomarker for this subtype [75]. Although typically not diagnosed at birth, affected individuals usually present with recurrent fractures after the first year of life, progressing to severe bone deformity, significant short stature, and a declining ability to walk independently [5, 73, 76] (Fig. 3). The hallmark of OI type VI is a profound mineralization defect characterized by the accumulation of unmineralized osteoid, reminiscent of osteomalacia, and a distinctive "fish-scale" pattern of lamellar bone under polarized light microscopy [5, 75].

Interestingly, a functional nexus exists between PEDF and the IFITM5 protein. A specific *IFITM5* mutation (c.119C > T, p.Ser40Leu) has been identified that phenotypically mimics OI type VI rather than the classic type V. Patients with this variant exhibit the characteristic "fish-scale" bone pattern and markedly reduced PEDF secretion from osteoblasts, despite maintaining normal serum PEDF levels [77]. While mechanistic studies indicate that BRIL and PEDF do not interact directly and are not involved in collagen processing, they appear to regulate osteoblast differentiation through complementary pathways [78]. The resulting genotype–phenotype correlation suggests that the severity of OI type VI is fundamentally tied to disruption of this PEDF-mediated mineralization axis. Patients often suffer from severely reduced BMD and eventual wheelchair dependence, illustrating the critical role of PEDF in the transition from osteoid deposition to mature bone formation [5, 77].

### Defects in post-translational modification of procollagen

Type I procollagen chains undergo essential post-translational modifications within the ER, including the hydroxylation of proline and lysine residues, which are critical for triple helix stabilization and supramolecular assembly [79] (Fig. 1b). A key enzymatic complex responsible for the 3-hydroxylation of Pro986 in the α1(I) chain consists of cartilage-associated protein (CRTAP), prolyl 3-hydroxylase 1 (P3H1), and cyclophilin B (CyPB), encoded by *CRTAP* (3p22**,** MIM 605497), *P3H1* (1p32, alternatively called *LEPRE* gene, MIM 610339), and peptidyl-prolyl isomerase B (*PPIB*) (15q22, MIM 123841), respectively [6–8, 36, 80]. Biallelic mutations in these genes disrupt the stability of this complex, leading to delayed collagen folding, enzymatic over modification, and impaired ECM formation. This molecular pathomechanism underlies a spectrum of severe to lethal autosomal recessive OI types (VII, VIII, and IX).

Clinically, these types exhibit significant phenotypic convergence due to functional interdependence of the complex subunits. OI type VII (*CRTAP,* MIM 610682) and OI type VIII (*P3H1,* MIM 610915) are characterized by perinatal fractures, extreme osteoporosis, rhizomelic limb shortening, and distinctive radiographic "popcorn" epiphyses [6, 7, 81]. While *CRTAP* mutations typically result in loss of function, some in-frame deletions produce dysfunctional proteins that fail to stabilize P3H1, leading to lethal outcomes [36, 80, 82, 83]. Similarly, *P3H1* deficiency results in almost absent 3-hydroxyproline at Pro986 (< 5%), with severity often dictated by specific alleles. For instance, the West African founder mutation (IVS5 + 1G > T) is associated with lethality in homozygotes [7, 84, 85]. OI type IX (*PPIB,* MIM 259440), while also involving 3-hydroxylation deficiency, presents a broader genotype–phenotype spectrum ranging from lethal to moderate forms [8]. Severe cases often involve homozygous frameshift or nonsense mutations, whereas some start-codon mutations (e.g., c.2T > G) result in milder phenotypes without rhizomelia [8, 86].

The genotype–phenotype correlation in these recessive forms is determined by the extent to which a mutation compromises the integrity of the entire P3H1/CRTAP/CyPB complex. Because each subunit is required for stability of the others, the complete absence of one often leads to the secondary depletion of its partners. However, the relatively milder spectrum in some *PPIB* mutations suggests that residual complex activity, facilitated by partially preserved P3H1 or CRTAP levels, may moderate disease severity by maintaining some level of peptidyl-prolyl isomerase or chaperone activity [8, 86]. Thus, the clinical expression of types VII–IX depends not only on the specific gene affected but on the functional residual capacity of the collagen-modifying machinery.

### Defects in procollagen processing, crosslinking, and intracellular trafficking

The protein products of serpin peptidase inhibitor, clade H, member 1 (*SERPINH1*, MIM 600943) and FK506-binding protein 10 (*FKBP10*, MIM 607063), heat shock protein 47 (HSP47) and FKBP65, respectively, function as essential ER-resident molecular chaperones. They prevent the premature formation of collagen protofibrils and stabilize the triple helix during its assembly and trafficking to the Golgi apparatus [79] (Fig. 1c).

#### Osteogenesis imperfecta type X (*SERPINH1*)

OI type X (MIM 613848) is an autosomal recessive disorder caused by biallelic mutations in *SERPINH1* (1p32). The hallmark molecular defect, such as the L78P missense mutation, does not alter initial post-translational modifications but impairs collagen quality by accelerating ER-to-Golgi transit. This produces structurally unstable, protease-sensitive collagen and disrupts the synergistic interaction between HSP47 and its partner FKBP65 [9].

The clinical spectrum ranges from moderate to perinatal-lethal forms [9, 87]. Patients typically present at birth with severe bone fragility, multiple fractures, generalized osteopenia, and long-bone bowing. Extraskeletal complications are a defining feature of type X, often including dentinogenesis imperfecta, blue or white sclerae, with additional systemic involvement including chronic lung disease, renal stones, or hydronephrosis [87].

Severity is largely dictated by the residual functional capacity of HSP47. Complete loss-of-function mutations often lead to respiratory failure and early childhood mortality due to severe thoracic restriction, whereas missense mutations may allow for survival into adulthood despite progressive skeletal deformities [9, 87].

#### Osteogenesis imperfecta type XI (*FKBP10*)

OI type XI (MIM 610968) arises from mutations in *FKBP10* (17q21), which encodes the chaperone FKBP65 [10]. Mechanistically, FKBP65 deficiency specifically impairs collagen cross-linking by preventing the hydroxylation of telopeptide lysines. This defect leads to significantly decreased collagen deposition into the ECM despite near-normal levels of secretion, resulting in profound bone fragility.

**Fig. 4:**
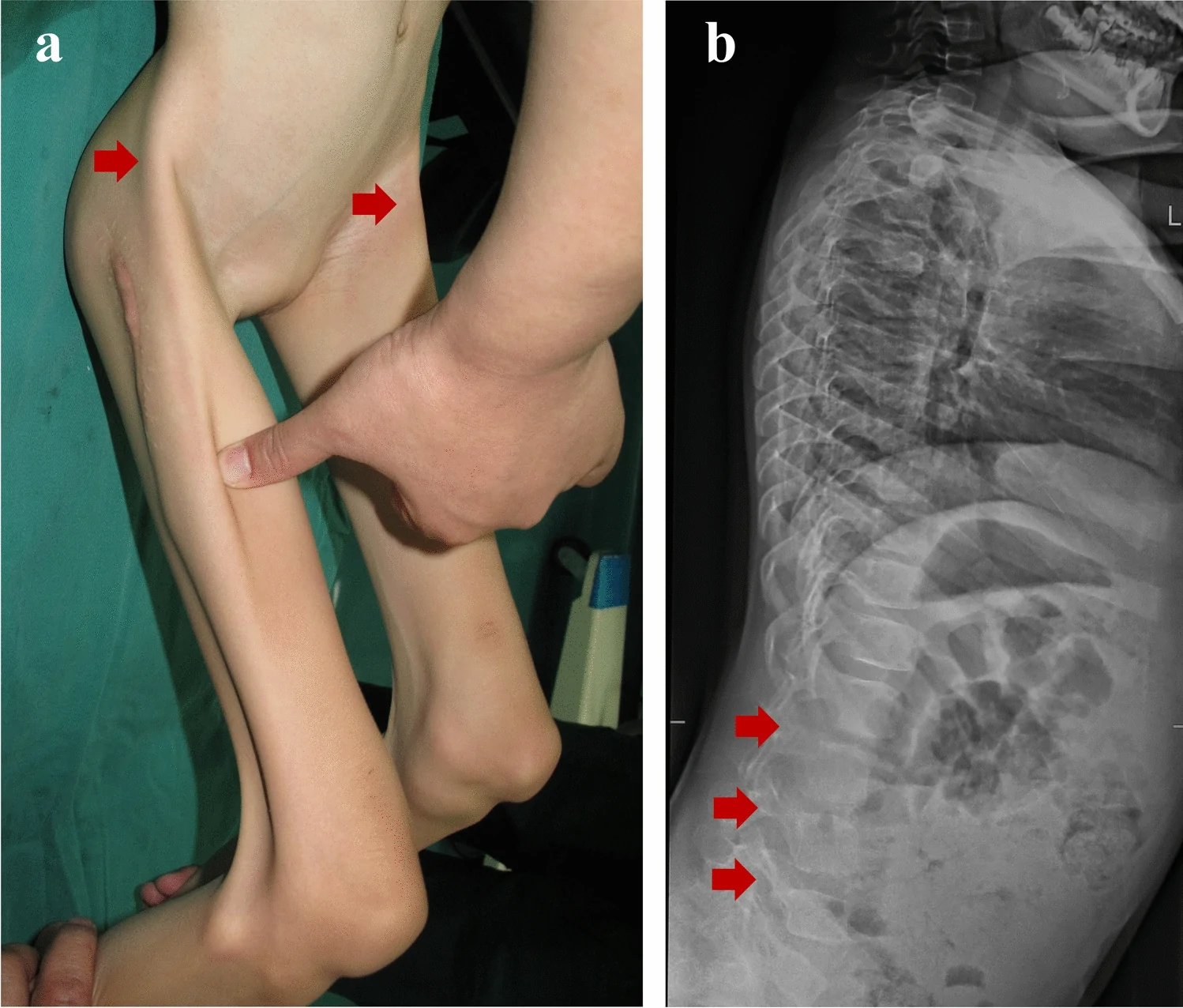
Characteristic manifestations in a patient with osteogenesis imperfecta type XI. **a** Congenital joint contractures of hip (red arrows); **b** lateral spinal view showing elongated pedicles (red arrows)

Patients primarily exhibit moderate to severe skeletal fragility, recurrent fractures in infancy, and progressive growth retardation [10, 88]. A distinguishing hallmark of OI type XI is the presence of variable congenital joint contractures, most commonly affecting the elbows and knees (Fig. 4). The phenotypic spectrum frequently overlaps with Bruck syndrome type I (BS1), where joint contractures and pterygia are predominant features [89].

Variability in *FKBP10*-related disorders suggests a continuum between classic OI and BS1. In some regions, such as Turkey, founder effects can lead to complex phenotypes, including co-inherited epidermolysis bullosa simplex [90]. While bisphosphonates may improve BMD, their efficacy in preventing the progression of skeletal deformities or joint contractures remains limited, particularly in BS1, highlighting a poor long-term functional prognosis for these patients [88–90].

#### Osteogenesis imperfecta type XIII (*BMP1*)

Following secretion, the transformation of procollagen into mature collagen fibrils requires the precise proteolytic cleavage of its N- and C-terminal propeptides. The metalloproteinase BMP1, also known as the major procollagen C-protease, is critical for the removal of the C-propeptide. Biallelic loss-of-function mutations in the *BMP1* gene (8p21.3, MIM 112264) impair this cleavage step, leading to defective type I procollagen processing and the assembly of abnormal, dysfunctional collagen fibrils [12] (Fig. 1c).

Clinically, OI type XIII (MIM 614856) presents a distinct profile that deviates significantly from the classical low-BMD form of collagen-related OI. The hallmark feature is a paradoxical combination of severe bone fragility, manifesting as recurrent long-bone and vertebral compression fractures from early childhood, with normal or even elevated BMD [10, 12]. Patients frequently exhibit delayed motor development, joint hypermobility, blue-grey sclerae, and umbilical hernias, while DI and severe limb deformities are notably less common [10]. Extraskeletal manifestations, such as muscle wasting, further distinguish this subtype [12].

Genotype–phenotype correlation in OI type XIII is primarily influenced by the impact of variants on different isoforms of the gene. Emerging evidence suggests that variants affecting both the shorter BMP1 and the longer mammalian tolloid (mTLD) isoforms are typically associated with more severe clinical presentations [91]. Conversely, mutations that partially preserve mTLD function may result in a relatively milder phenotype. Whilest specific variant location within the functional domains of *BMP1* [e.g., the protease or complement C1r/C1s, Uegf, and Bmp1 (CUB) domains] has been studied, it does not yet show a clear predictive correlation with specific clinical features. This indicates that overall residual activity of the C-protease complex is the primary determinant of disease severity [91, 92].

#### Osteogenesis imperfecta type XXI (*KDELR2*)

OI type XXI (MIM 619131) is a recently identified autosomal recessive disorder resulting from biallelic mutations in the KDEL endoplasmic reticulum protein retention receptor 2 (*KDELR2)* gene (MIM 609024) [20]. The KDEL receptor 2 is essential for the retrograde transport of ER-resident proteins from the Golgi apparatus back to the ER. Molecular studies have revealed that *KDELR2* mutations impair the ability of the receptor to bind and recycle the collagen chaperone HSP47. Consequently, HSP47 fails to be retrieved and remains associated with procollagen as it moves into the ECM. This persistent association disrupts normal fibrillogenesis and collagen maturation, establishing OI type XXI as a unique trafficking disorder where the failure of chaperone retrieval compromises bone matrix quality [20].

Clinically, OI type XXI is characterized by a progressively deforming skeletal disorder with severe bone fragility. The cardinal features include multiple fractures, often occurring prenatally or in early childhood, leading to progressive skeletal deformities, disproportionate short stature, scoliosis, and characteristic chest deformities (barrel-shaped or bell-shaped) [20]. The disease course is typically severe, with many patients becoming wheelchair-bound by adolescence. Whilst early reports emphasized the absence of classic features like blue sclerae and DI, subsequent clinical observations have identified these findings in a subset of patients. Of note, the phenotypic spectrum extends beyond the skeletal system to include neurodevelopmental features, such as hypotonia and delays in motor and speech development, suggesting a broader systemic impact of *KDELR2* dysfunction on the central nervous system [93].

Genotype–phenotype correlation in OI type XXI reflects the critical threshold in HSP47 retrieval. Total or near-total loss of *KDELR2* function appears to correlate with the most severe progressively deforming phenotypes and systemic complications. Furthermore, the limited efficacy of bisphosphonate therapy observed in these patients underscores the profound nature of the underlying matrix assembly defect, which differs mechanistically from the primary mineralization or collagen synthesis issues seen in more common OI types [20, 93].

### Genetic defects in osteoblast differentiation and homeostasis

#### Osteogenesis imperfecta type XII (*SP7*)

The specificity protein 7 (*SP7)*-related form of OI (OI type XII; MIM 613849) is a distinct skeletal dysplasia caused by mutations in the *SP7* gene (MIM 606633). *SP7*, also known as *Osx*, encodes the zinc-finger transcription factor Osterix (Fig. 1e). Within the osteogenic transcriptional hierarchy, SP7 acts downstream of runt-related transcription factor 2 (RUNX2) and is indispensable for the differentiation of mesenchymal progenitors into functional, bone-forming osteoblasts. Pathogenically, a loss of *SP7* function effectively halts osteoblast lineage maturation. This mechanism has been elucidated in mouse models where Osx deficiency results in a total failure of bone formation despite preserved cartilage development and normal *RUNX2* expression [44]. Consequently, SP7 serves as a critical molecular switch for specifying osteoblast lineage during skeletogenesis.

Clinically, OI type XII was first genetically characterized in 2010 through homozygosity mapping in an Egyptian patient harboring a homozygous frameshift mutation [11]. The clinical spectrum is primarily defined by moderate-to-severe bone fragility, recurrent fractures, long bone deformities, and significant growth deficiency. Of note, patients with *SP7*-related OI typically present with normal sclerae and lack the DI common in other OI types. Sensorineural hearing loss has emerged as a significant, albeit variable, extraskeletal feature, as highlighted by reports of affected Iraqi siblings carrying the p.Arg316Cys variant [94].

The correlation between genotype and phenotype in *SP7*-related OI is remarkably distinct, dictated by the inheritance pattern and the nature of the molecular defect. Biallelic loss-of-function mutations, such as the frameshift c.1052delA (p.E351GfsTer19), follow an autosomal recessive inheritance pattern and manifest as a classical severe bone fragility phenotype [11, 94]. Conversely, a specific de novo neomorphic missense mutation (p.S309W) results in an autosomal dominant form of the disease. This unique mutation alters transcription factor DNA-binding specificity, leading to a complex high-turnover bone disorder. This phenotype is characterized by a paradoxical combination of skeletal fragility and defective mineralization alongside osteosclerotic features, including cranial hyperostosis and craniosynostosis [95].

#### Osteogenesis imperfecta type XV (*WNT1*)

OI type XV (MIM 615220) is an autosomal recessive disorder caused by biallelic pathogenic variants in the wingless-type MMTV integration site family, member 1 (*WNT1*) gene, which encodes a critical ligand in the canonical Wnt/β-catenin signaling pathway. The *WNT1* gene plays a pivotal role in skeletal homeostasis by regulating osteoblast differentiation and function. Mutant *WNT1* proteins fail to properly activate the canonical signaling cascade, thereby compromising the nuclear translocation of β-catenin and subsequent transcriptional activation of osteogenic genes. Functional studies have demonstrated that these mutant proteins significantly impair Wnt signaling activity and disrupt osteoblast differentiation in vitro [14, 96]. First reported simultaneously in 2013 by three independent research groups [14, 48, 96], this condition is now recognized as the most common autosomal recessive form of OI in several populations, including China [97].

**Fig. 5:**
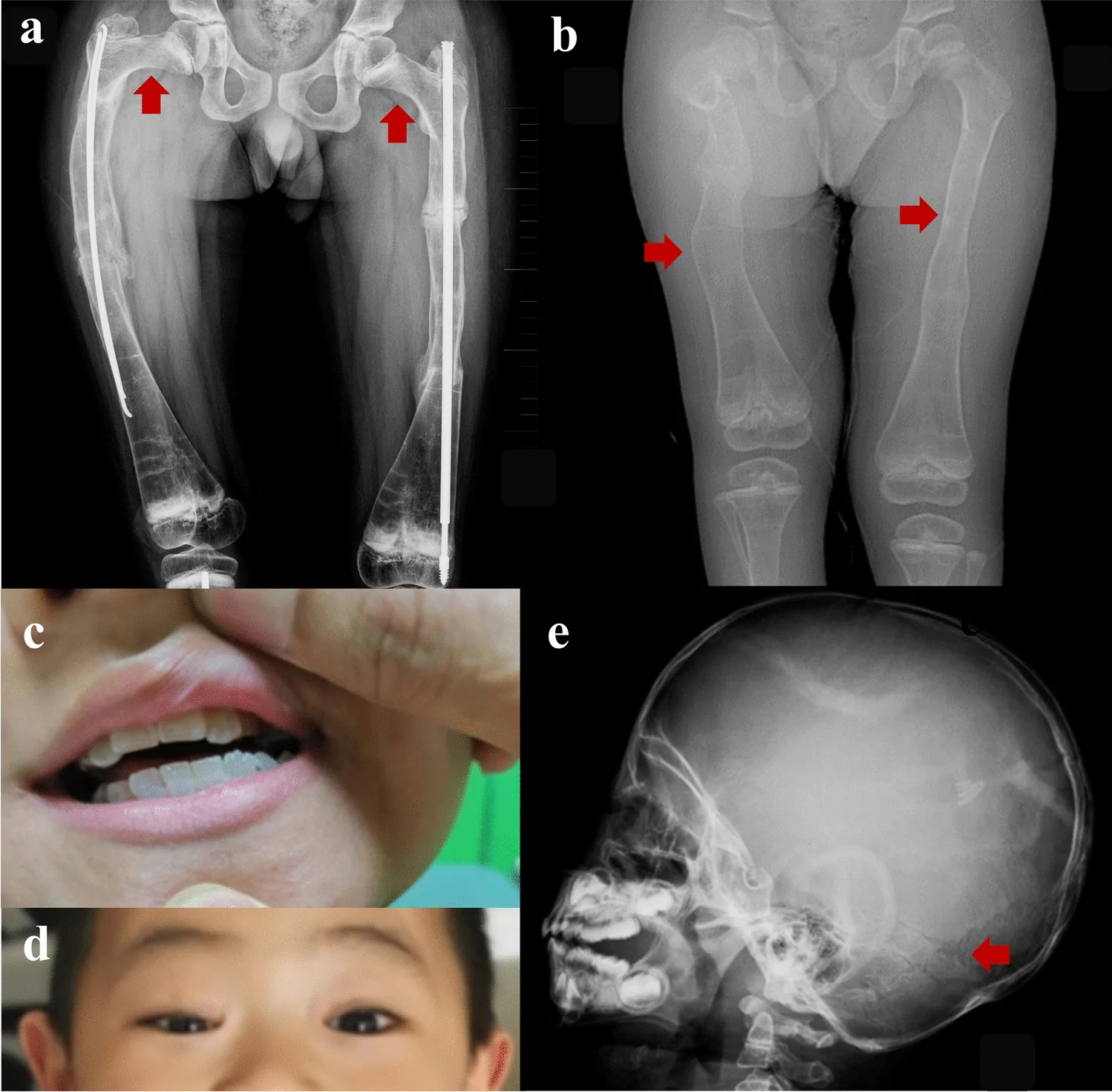
Clinical features of patients with osteogenesis imperfecta type XV. **a** Radiograph of the pelvis and femurs showing bilateral coxa vara; **b** radiograph of the lower limbs with thin bone cortices and bowing of the femurs; **c** dentinogenesis imperfecta; **d** facial photograph of a patient showing bilateral ptosis; **e** lateral skull radiograph showing multiple wormian bones along the cranial sutures

Clinically, the presentation of OI type XV is characterized by significant phenotypic heterogeneity, ranging from moderate to progressively deforming skeletal fragility [14]. Key manifestations include early-onset recurrent fractures, long bone bowing, reduced calvarial mineralization, scoliosis, and short stature. Common extraskeletal features include blue-gray sclerae, ligamentous laxity, and, in some cases, DI [98, 99]. A hallmark of OI type XV that differentiates it from other forms is the high prevalence of neurological involvement. Recessive *WNT1*-related OI predominantly affects the brainstem and cerebellum, often leading to brainstem-cerebellar hypoplasia and developmental delay [100]. Of note, unilateral or bilateral ptosis is observed in approximately 40% of reported cases, serving as a vital clinical indicator for targeted molecular diagnosis [97] (Fig. 5).

The clinical manifestations of *WNT1*-related disorders are strongly influenced by whether pathogenic variants are present in a monoallelic or biallelic state. Biallelic homozygous or compound heterozygous mutations result in the severe, recessive OI type XV phenotype described above, characterized by profound skeletal and often neurological impairment [97]. In contrast, heterozygous carriers typically exhibit normal linear growth and lack long-bone deformities; however, they are predisposed to a milder skeletal phenotype. These individuals may demonstrate reduced BMD, early onset osteoporosis, or isolated fractures, highlighting the crucial requirement for balanced Wnt1 signaling in maintaining lifelong bone mass [14, 96].

#### Osteogenesis imperfecta type XX (*MESD*)

OI type XX (MIM 618644) is an autosomal recessive skeletal disorder caused by biallelic mutations in the mesoderm development LRP chaperone (*MESD*) gene (MIM 607783). The genetic basis of this condition was established by Moosa et al. in 2019, who identified homozygous loss-of-function variants in multiple affected families [19]. The pathogenic mechanism centers on the role of *MESD* as a specialized chaperone within the ER that is specifically required for the proper folding and trafficking of the Wnt co-receptors low density lipoprotein receptor-related protein 5 (LRP5) and LRP6. Pathogenic mutations, particularly those leading to a loss of the ER-retention domain, impair the intracellular localization and function of MESD. This disruption prevents LRP5/6 from reaching the cell surface, thereby compromising the canonical Wnt signaling pathway, a cascade essential for osteoblast differentiation, function, and bone mineralization.

Clinically, OI type XX is a severe, progressively deforming disorder characterized by profound osteopenia and recurrent fractures, which often occur prenatally or in early infancy [19]. The phenotypic spectrum is remarkably broad, ranging from lethal perinatal forms involving multiple intrauterine fractures and hydrops to a childhood-onset form marked by severe skeletal deformities, such as scoliosis and a narrowed thorax. The latter can lead to lung hypoplasia and fatal respiratory failure [19]. Distinctive clinical features include blue sclerae during infancy and a specific facial phenotype characterized by a triangular face and retrognathia. While classic DI is typically absent, patients may exhibit oligodontia or disorganized dentition. In some instances, global developmental delay has been observed [101].

#### Osteogenesis imperfecta type XVI (*CREB3L1*)

OI type XVI is an autosomal recessive disorder caused by biallelic mutations in the cAMP response element-binding protein 3-like 1 (*CREB3L1*) gene, which encodes the ER-stress transducer old astrocyte specific induced substance (OASIS, Fig. 1f). First linked to OI in 2018 through exome sequencing, this gene plays a critical role in the capacity of osteoblasts to handle high-volume collagen synthesis [15]. The underlying pathogenic mechanism involves a failure in the coat protein-II-mediated (COPII) secretory pathway. Specifically, OASIS acts as a transcription factor that regulates the expression of *SEC24D*, a key component of the COPII complex required for the transport of procollagen from the ER to the Golgi apparatus. Mutations in *CREB3L1* abolish the DNA-binding capacity of OASIS, leading to defective expression of member D of SEC24-related gene family (*SEC24D*) and the subsequent retention of collagen within the ER lumen. This sequestration results in pathological ER expansion and compromised bone matrix formation, findings consistent with *Oasis* knockout mice, which display impaired osteoblast maturation and intracellular collagen accumulation. Thus, *CREB3L1* mutations drive the OI phenotype by disrupting the protein trafficking essential for bone extracellular matrix integrity.

The clinical spectrum of OI type XVI is notably heterogeneous, exhibiting a broad range of severity that correlates with impact of the mutation on protein function. Biallelic loss-of-function variants in *CREB3L1* result in a phenotype ranging from perinatal lethality to a progressively deforming, severe type III-like OI compatible with survival into childhood [15]. The most severe manifestations are typically observed in individuals with complete loss of OASIS function, such as those with homozygous whole-gene deletions or protein-truncating variants. This results in a lethal prenatal form (resembling OI type II) characterized by extreme bone fragility, in utero fractures, severe micromelia, beaded ribs, and marked calvarial under-mineralization, leading to perinatal death from thoracic insufficiency. In contrast, heterozygous carriers of these null variants are typically asymptomatic or exhibit negligible clinical signs, such as blue sclerae, without a significant fracture history [102].

A distinct intermediate phenotype, characterized by a severe but non-lethal clinical course (resembling OI type III), is associated with hypomorphic variants that allow for residual protein function. For example, a patient homozygous for a premature stop codon (p.Tyr428Ter), which likely undergoes incomplete nonsense-mediated decay, was reported to survive into the second decade of life [103]. His clinical course included severe, progressive skeletal deformity, recurrent fractures, short stature, blue sclerae, and tooth agenesis. Interestingly, while heterozygous carriers in some families are clinically unaffected, others carrying specific missense mutations or in-frame deletions may display mild OI features, including reduced bone mineral density and occasional fractures. This suggests a complex genotype–phenotype correlation where certain qualitative variants may exert a dominant-negative effect, contrasting with the purely recessive nature of true null alleles.

#### Osteogenesis imperfecta type XIX (*MBTPS2*)

OI type XIX (MIM 301014) is a distinct X-linked recessive disorder caused by mutations in the membrane-bound transcription factor protease, site 2 (*MBTPS2*) gene (MIM 300294). This gene encodes the site-2 protease (S2P), a zinc metalloprotease essential for the intramembrane cleavage of transcription factors. The genetic etiology of this subtype was first elucidated by Lindert et al. in 2016 through the identification of two missense mutations, p.N459S and p.L505F, in unrelated families presenting with moderate-to-severe skeletal fragility [18]. The primary pathogenic mechanism involves disruption of regulated intramembrane proteolysis (RIP), whereby mutant *MBTPS2* impairs the proteolytic activation of key ER-stress transducers and transcription factors, most notably OASIS and activating transcription factor 6 (ATF6). These factors are indispensable for normal ER stress responses and the specialized differentiation of osteoblasts. Consequently, cells derived from affected patients exhibit broad defects in osteoblast maturation and a significant reduction in the secretion of type I collagen. Furthermore, biochemical analysis of bone collagen has revealed abnormal lysine hydroxylation, a modification critical for proper cross-linking and bone tensile strength. Thus, *MBTPS2* mutations compromise skeletal integrity through a dual mechanism: attenuation of collagen trafficking and modification of essential post-translational modifications.

The clinical phenotype of OI type XIX is characterized by a moderate-to-severe skeletal presentation with a typical X-linked inheritance pattern. Affected individuals often present with prenatal or early-onset fractures of the ribs and long bones, accompanied by generalized osteopenia and severe short stature in adulthood [18, 104]. Prominent radiographic manifestations include progressive bowing of the long bones, specifically the humeri, radii, and tibiae, as well as kyphoscoliosis, anterior vertebral wedging, and a characteristic marked anterior angulation of the tibia. Thoracic cage abnormalities, such as pectus carinatum or excavatum, are also frequently observed. Interestingly, while the scleral hue may be blue during childhood, it often transitions to white in adulthood. A notable aspect of the genotype–phenotype correlation in *MBTPS2* mutations is that while the gene is also associated with dermatological syndromes like ichthyosis follicularis, atrichia, and photophobia (IFAP), patients with OI type XIX remarkably lack these cutaneous features (e.g., ichthyosis or alopecia). This suggests that the specific missense variants identified in OI type XIX selectively impair the signaling pathways required for bone homeostasis while sparing those critical for epidermal development.

#### Osteogenesis imperfecta type XVII (*SPARC*)

OI type XVII (MIM 616507) is caused by biallelic loss-of-function mutations in the secreted protein, acidic, cysteine-rich (*SPARC*) gene (8q23.1), which encodes osteonectin, a multifunctional secreted glycoprotein abundant in the ECM. SPARC plays a critical role in bone remodeling by supporting the maintenance of bone mass through the regulation of osteoblast and osteoclast activity. At the molecular level, SPARC acts as a chaperone that binds to type I collagen and hydroxyapatite, facilitating procollagen processing and collagen fibrillogenesis [105]. In 2015, Mendoza-Londono et al. first identified homozygous missense mutations (p.Arg166His and p.Glu263Lys) that disrupt a critical intramolecular salt bridge within the collagen-binding domain [16]. This defect leads to delayed collagen secretion and mild triple-helical over modification. Furthermore, as demonstrated in *SPARC*-null mouse models, the absence of functional SPARC results in decreased osteoblast numbers and impaired bone formation, leading to severe bone loss and a negative bone balance.

Clinically, *SPARC*-related OI presents as a severe bone fragility disorder characterized by recurrent long bone and vertebral compression fractures starting in infancy [16]. A unique neuromuscular presentation, including profound muscle weakness, hypotonia, and delayed motor development, is often the initial hallmark and may lead to misdiagnosis of primary myopathy [106, 107]. Radiographic findings typically show thin cortices and slender diaphyses, while bone analysis reveals hypermineralization. Other features include progressive early-onset scoliosis, short stature, joint hyperlaxity, and occasionally blue-grey sclerae or neurodevelopmental delays.

#### Osteogenesis imperfecta type XIV (*TMEM38B*)

OI type XIV (MIM 615066) is an autosomal recessive disorder caused by biallelic mutations in transmembrane protein 38B (*TMEM38B*), which encodes the trimeric intracellular cation channel subtype B (TRIC-B) (Fig. 1g). This gene was initially identified through genetic linkage and exome sequencing in consanguineous families, with many cases attributed to a recurrent homozygous truncating mutation (c.455_542del) [13, 46]. The molecular pathogenesis of OI type XIV centers on the disruption of ER calcium dynamics. TRIC-B serves as a monovalent cation channel that provides a counter-flux to balance the charge during calcium release from the ER. Consequently, the loss of TRIC-B, as demonstrated in *Tricb* knockout murine models, impairs the mobilization of ER calcium stores [47]. In osteoblasts, this dysregulation triggers chronic ER stress and leads to the intracellular sequestration of collagen, ultimately resulting in a failure of proper collagen secretion into the bone matrix [108, 109]. This defect in collagen trafficking and the subsequent impairment of mineralization underpin the skeletal fragility observed in these patients.

Clinical manifestations of OI type XIV encompass a broad phenotypic spectrum, ranging from near-asymptomatic presentations to severe skeletal deformity [13, 46]. The core clinical features include recurrent fractures with an onset ranging from the prenatal period to the first six years of life, alongside generalized osteopenia and low BMD. Radiographic findings are often characterized by vertebral compression fractures, coxa vara, and "periosteal cloaking", a distinctive feature involving the deposition of new bone beneath the periosteum. Of note, and in contrast to classical collagen-related OI, patients with OI type XIV typically present with normal sclerae, dentition, and hearing. However, extraskeletal involvement has been documented in a subset of individuals, including muscular hypotonia and cardiac abnormalities such as septal hypertrophy and valvular regurgitation, likely reflecting the systemic impact of disrupted calcium signaling. Histomorphometric analysis of bone tissue reveals a distinct low-turnover state, marked by significantly reduced osteoblast and osteoclast activity. Despite this low-turnover profile, patients generally exhibit a favorable response to bisphosphonate therapy, achieving significant increases in BMD and clinical stability [110, 111].

### Emerging osteogenesis imperfecta genes and novel candidates with uncertain pathogenicity

#### Osteogenesis imperfecta type XVIII (*TENT5A*)

OI type XVIII (MIM 617952) is a severe autosomal recessive disorder caused by biallelic loss-of-function mutations in the terminal nucleotidyltransferase 5A (*TENT5A*) gene (formerly *FAM46A*, MIM 611357) (Fig. 1f). This gene encodes a cytoplasmic nucleotidyltransferase that serves as a terminal adenylyltransferase, playing a critical role in the polyadenylation and subsequent stability of specific mRNA transcripts. The genetic basis of this subtype was first established by Doyard et al. in 2018 through exome sequencing in affected individuals from three unrelated consanguineous families [17]. While specific bone-related mRNA targets of *TENT5A* remain to be fully elucidated, the primary pathogenic mechanism involves disrupted mRNA regulation, which adversely affects the translation of proteins essential for osteoblast function and bone matrix formation. The critical nature of this gene in skeletal development is further shown by mouse models that recapitulate the human phenotype, demonstrating extreme bone fragility, spontaneous fractures, and significantly elevated alkaline phosphatase activity.

Clinically, OI type XVIII represents an exceptionally severe form of the disease, often presenting with extreme bone fragility and multiple fractures at birth. Core clinical manifestations include a high annual fracture rate beginning in infancy and a hallmark feature of congenital bowing of the long bones, particularly the femora and tibiae. Radiographic findings are consistent with severe generalized osteopenia and include thin cortical bone, the presence of wormian bones, and multiple vertebral collapses [112]. Common extraskeletal features frequently observed in these patients include blue sclerae and systemic joint hyperlaxity. Furthermore, significant delays in motor and speech development are often reported, adding a neurological dimension to the clinical burden. The phenotype is frequently severe enough to be perinatally lethal in some instances. Of note, the initial clinical presentation, characterized by congenital bowing and, occasionally, transient dysautonomia symptoms such as hyperthermia, may lead to diagnostic overlap with Stüve-Wiedemann syndrome. However, the progressive clinical course and radiographic hallmarks eventually distinguish OI type XVIII as a primary defect in bone development and mineralization.

#### Osteogenesis imperfecta type XXII (*CCDC134*)

OI type XXII (MIM 619795) is an autosomal recessive disorder caused by biallelic loss-of-function mutations in the coiled-coil domain-containing protein 134 (*CCDC134*) gene. A recurrent homozygous mutation in the initiation codon (c.2T > C, p.M1T), first identified by Dubail et al. in 2020, represents a founder variant frequently reported in patients of Moroccan and Brazilian ancestry [21]. Pathogenically, *CCDC134* acts as a secreted signaling regulator that is essential for maintaining balanced intracellular cascades during osteoblast differentiation. Its loss leads to dysregulation of the RAS/MAPK pathway, characterized by pathologically elevated extracellular signal-regulated kinase (ERK) 1/2 phosphorylation in patient-derived osteoblasts. This aberrant signaling environment compromises osteoblast function, resulting in the markedly reduced expression of key bone matrix components, such as *COL1A1* and osteopontin, and severely impaired mineralization. The fundamental requirement for CCDC134 in skeletal development is further underscored by the embryonic lethality and profound developmental abnormalities observed in *Ccdc134*-null murine models.

Clinically, OI type XXII is characterized by extreme bone fragility and a broad phenotypic spectrum that ranges from perinatal lethality to severe lifelong disability [21, 113, 114]. Affected individuals frequently present with multiple prenatal or early-onset fractures of the long bones and ribs, which often progress to pseudarthrosis. Growth is severely impacted, with significant short stature often preceded by intrauterine growth restriction. Radiographic findings typically include the presence of wormian bones, gracile and bowed long bones with low mineral density, and progressive scoliosis. Distinguishing clinical features of this subtype include a consistent absence of DI and sclerae that are typically white or slightly grey, rather than the blue hue seen in classical collagen-related forms. Patients are also prone to serious complications, such as complex spinal deformities and craniovertebral junction abnormalities, including basilar invagination, which often necessitate repeated and challenging orthopedic surgeries.

#### Osteogenesis imperfecta type XXIII (*PHLDB1*)

OI type XXIII (MIM 620639) represents a recently characterized mild autosomal recessive form of the disease, resulting from biallelic loss-of-function mutations in the pleckstrin homology-like domain, family B, member 1 (*PHLDB1*) gene (MIM 612834) located on chromosome 11q23 [115]. The gene encodes a protein characterized by a critical C-terminal pleckstrin homology (PH) domain, which specifically interacts with phosphatidylinositol 3,4,5-trisphosphate [PtdIns(3,4,5)P_3_]. This interaction is essential for insulin-dependent protein kinase B (AKT) phosphorylation, a signaling axis fundamental to bone cell homeostasis. Identified pathogenic variants, specifically frameshift mutations such as c.2392dup and c.2690_2693del, lead to premature termination codons and the resultant loss of the C-terminal PH domain. This truncation disrupts the phosphoinositide-mediated signaling cascades required for robust osteogenic differentiation and collagen biosynthesis, thereby precipitating the characteristic skeletal fragility and mineralization defects of this subtype [22].

Clinically, OI type XXIII is distinguished by a relatively mild phenotype with a unique regressive clinical course [22]. The hallmark presentation during early childhood involves recurrent fractures, generalized osteopenia, and radiographic evidence of platyspondyly, widened metaphyses, and shortened, bowed long bones. A defining feature of the genotype–phenotype correlation in *PHLDB1* mutations is the transient nature of several skeletal findings; the metaphyseal and vertebral abnormalities often demonstrate significant spontaneous improvement after early childhood. While the underlying osteopenia tends to persist, it typically shows a favorable response to bisphosphonate therapy. Extraskeletal manifestations may include blue or grey sclerae and mild motor delays. Of note, some patients develop late-onset truncal obesity, which may reflect the role of PHLDB1 in insulin signaling and systemic metabolic regulation beyond its primary impact on the skeleton.

## Epidemiological and clinical characterization of osteogenesis imperfecta in Asian populations

### China

Comprehensive characterization of the phenotypic and genotypic landscapes of Chinese OI patients have been established by three leading research groups [73, 116–119]. The majority of OI cases in China are classical autosomal dominant forms resulting from defects in type I collagen. Variants in *COL1A1* and *COL1A2* account for 76%–90% of molecular diagnoses, with *COL1A1* mutations being more prevalent (44%–58%) than *COL1A2* (29%–42%).

Based on the Sillence classification, the Chinese cohort exhibits a distribution pattern where type I (mild) remains the most frequent subtype (39%–51%), followed by type IV (29%–35%) and type III (about 20%) [73, 116, 117]. Type V OI, exclusively associated with the *IFITM5* c.-14C > T recurrent mutation, accounts for approximately 3%–6% of cases. Clinical presentation in these cohorts is near-universal recurrent fractures and a median age at onset of 3 years. While blue sclera is a hallmark of classical collagen-related OI (81%–93%), it is markedly less prevalent in recessive forms (about 40%). Conversely, skeletal severity is more pronounced in adult cohorts, where long bone deformities and vertebral compression fractures affect 92% and 80%–100% of type III/IV patients, respectively. DI remains a significant comorbidity, particularly in more severe structural collagen defects. Genetic mapping has identified several recurrent mutations that suggest either high-frequency hotspots or potential founder effects within the Chinese population. In the collagen genes, *COL1A1* variants c.769G > A (p.Gly257Arg) and c.2461G > A (p.Gly821Ser), and the *COL1A2* variant c.1009G > A (p.Gly337Ser) are frequently detected across unrelated pedigrees.

A distinguishing feature of the Chinese OI population is the unique genetic spectrum of its recessive forms [118, 119]. While Western cohorts often report a higher prevalence of mutations in the prolyl 3-hydroxylation complex (e.g.,* CRTAP, P3H1*), Chinese autosomal recessive OI families show a distinct predilection for *WNT1* mutations. In a comprehensive analysis of 74 recessive OI families, *WNT1* emerged as the most frequent causative gene (40.54%), followed by *SERPINF1* (29.7%) and *FKBP10* (13.5%). Phenotypically, Chinese recessive OI patients typically present with a worse clinical course than those with classical dominant OI. This includes significantly lower BMD *Z*-scores and a higher incidence of severe skeletal deformities (80%). Of note, *WNT1*-related OI in China has been associated with specific extraskeletal markers, such as ptosis, which may serve as a clinical sentinel for this genotype.

### South Korea

In the Korean population, defects in *COL1A1* and *COL1A2* genes account for approximately 87.4% of genetically confirmed OI cases [120, 121]. Among these, *COL1A1* variants are the most prevalent, identified in 66.7% of probands, while *COL1A2* variants are found in 20.7%. Clinical categorization based on the Sillence classification system reveals that type I (mild) is the most frequent phenotype, representing 54.5% of the Korean cohort, followed by type IV (moderate, 21.2%), type III (severe, 16.2%), and type V (8.1%). Genotype–phenotype correlations indicate that glycine substitution variants result in significantly more severe clinical manifestations compared to other variants, including a shorter stature (mean height *Z*-score of −5.94 vs. −1.66) and an earlier onset of initial fractures (11.12 months vs. 23.74 months). Furthermore, 62.5% of patients with glycine substitution over the age of 14 experienced more than 10 lifetime fractures. Non-skeletal features also exhibit a gene-specific distribution; blue sclera is observed in the vast majority of *COL1A1* variant carriers (93.4%) but only in 43.8% of those with *COL1A2* variants. DI and wormian bones are also highly prevalent in the glycine substitution group, occurring in 71.1% and 89.1% of cases, respectively.

Autosomal recessive forms of OI are notably rare in South Korea, comprising only 4.0% of total cases. This is attributed to the strong sociocultural taboo against consanguineous marriage in the region [120, 121]. The most frequently identified recessive OI variants involve the *FKBP10* gene (3.0%), with other rare cases involving *BMP1* and *WNT1*. This distribution differs from populations with high rates of consanguinity, where recessive OI genes involved in post-translational modifications, such as *CRTAP* and *P3H1*, are more commonly reported. Regarding molecular hotspots and founder effects, several recurrent mutations have been identified within the Korean population. Notably, the *COL1A2* c.982G > A (p.Gly328Ser) mutation has been identified as a significant recurrent variant found in five independent probands. Haplotype analysis revealed that four of these five probands shared a common haplotype, providing strong evidence for a founder mutation in the Korean population. Other identified recurrent variants include *COL1A1* c.2089C > T (p.Arg697*) and c.2461G > A (p.Gly821Ser), which further define the unique mutational spectrum of OI in the south Korea.

### Japan

In the Japanese population, pathogenic variants in the *COL1A1* and *COL1A2* genes account for approximately 90% of genetically confirmed OI cases [122–124]. Recent comprehensive genetic screening utilizing next-generation sequencing (NGS) and Sanger sequencing has identified *COL1A1* mutations as the most prevalent (in 66.7%–69.8% of patients), followed by *COL1A2* mutations (20.7%–27.9%). Clinical distribution according to the Sillence classification indicates a predominance of type I (mild), representing 54%–56% of cohorts. Within these collagen genes, specific triple-helical glycine substitutions, notably *COL1A1* p.Gly821Ser and *COL1A2* p.Gly334Ser, have been identified as recurrent variants. In addition, OI type V accounts for approximately 2.3%–8.1% of cases in specific Japanese studies. Conversely, recessive OI is significantly less frequent in Japan than in regions with higher rates of consanguinity. In Japanese cohorts, variants in genes such as *FKBP10*, *BMP1*, and *WNT1* have been reported, though they represent only a small fraction of the total OI population.

### Vietnam

Molecular analysis of 91 unrelated Vietnamese patients reveals a unique genetic landscape for OI [125]. While *COL1A1* (36.3%) and *COL1A2* (23.1%) mutations remain the primary variants, their combined prevalence of 59.4% is significantly lower than the 85%–90% typically observed in Western and other Asian cohorts, suggesting a higher contribution of non-collagenous factors. Within these classical cases, *COL1A1* variants c.2461G > A (p.Gly821Ser) and c.2005G > A (p.Ala669Thr) emerge as frequent genotypes.

Of note, recessive OI is disproportionately prevalent in Vietnam, accounting for at least 11.6% of cases [126]. Type VIII OI, driven by *P3H1* mutations, is the most common recessive form, followed by variants in *CRTAP* and *FKBP10*. Unlike the severe or lethal recessive phenotypes usually reported globally, the Vietnamese cohort displays a broader clinical spectrum, including the first documented case of mild OI linked to *P3H1*. Central to this profile is the *P3H1* splice-site mutation c.1170 + 5G > C, which was identified in 85.7% of recessive families and 10.3% of the total OI population. Its high prevalence within the Kinh ethnicity establishes it as a critical founder-like molecular marker for targeted screening and genetic counseling in Vietnam.

### India

The molecular architecture of OI in India diverges significantly from other cohorts, characterized by an attenuated prevalence of classical collagenous variants and a pronounced enrichment of recessive forms. In a major Indian referral center study, heterozygous *COL1A1* and *COL1A2* variants accounted for only 47.6% of cases, substantially lower than the 85%–90% global benchmark [127]. Clinically, the cohort tended towards greater severity; Sillence type III was most prevalent (44%), followed by type IV (32%) and type I (24%). This severity is reflected in the high incidence of limb deformities (90% lower limb and 39% upper limb) and early-onset fractures, often occurring in utero or within the first six months of life. Extraskeletal presentations were equally prominent, with blue sclerae and DI occurring in 84% and 47% of patients, respectively. Of note, the unusually high frequency of recessive OI (up to 48%) is likely driven by regional consanguinity [128, 129]. Mutations in *P3H1* and *FKBP10* were particularly frequent, and the identification of recurrent variants, such as *WNT1* (c.506dupG) and *FKBP10* (c.1346-1G > C), across unrelated families strongly suggests potential founder effects within specific subpopulations.

## Current strategies for the molecular diagnosis of osteogenesis imperfecta

In a clinical setting, the diagnostic approach for OI must be strategically tailored to the phenotype of the patient, balancing cost-effectiveness with the need to identify both common and rare variants across 35 associated genes [3]. While variants in *COL1A1* and *COL1A2* are the primary genetic targets, clinicians must account for a broad spectrum of genetic alterations, including single nucleotide variants, small indels, and complex structural rearrangements [130–133]. NGS, specifically gene panels or whole exome sequencing (WES), is now the recommended first-line diagnostic tool. However, to avoid diagnostic gaps, clinicians should ensure that WES protocols include dedicated copy number variant analysis and structural variant algorithms (such as Manta) to capture exon-level deletions or duplications that standard pipelines might miss.

When initial NGS results are negative or inconclusive despite a suggestive phenotype, the decision-making process should pivot toward specialized techniques. Karyotyping remains a critical diagnostic consideration for detecting balanced chromosomal translocations; if a rearrangement is identified in a region harboring OI genes, the clinician should escalate to whole genome sequencing (WGS) or long-read sequencing to precisely map the breakpoints [134]. Long-read sequencing is particularly effective as a supplementary tool for resolving complex genomic structures that confound short-read technologies [130]. Conversely, the use of Sanger sequencing should be restricted to high-specificity clinical scenarios, such as the rapid confirmation of the *IFITM5* c.-14C > T variant in suspected type V OI or the targeted screening of known familial variants.

Final clinical determination relies on a rigorous interpretation of variants following American College of Medical Genetics and Genomics (ACMG)/Association for Molecular Pathology (AMP) and Clinical Genome Resource (ClinGen) guidelines, often incorporating collagen-specific criteria [135]. To resolve variants of uncertain significance, clinicians should utilize auxiliary functional assays. For suspected splicing defects, RNA analysis, using either traditional skin fibroblasts or non-invasive urine-derived cells, and minigene reporter assays provide essential evidence of pathogenicity [136–138]. Furthermore, collagen electrophoresis remains a vital diagnostic adjunct, offering a functional readout of type I collagen synthesis that can provide the definitive biochemical evidence needed to confirm a diagnosis when molecular findings are ambiguous.

## Targeted therapies for osteogenesis imperfecta: tailoring treatment to genetic etiology

### Orthopedic and surgical interventions

**Fig. 6:**
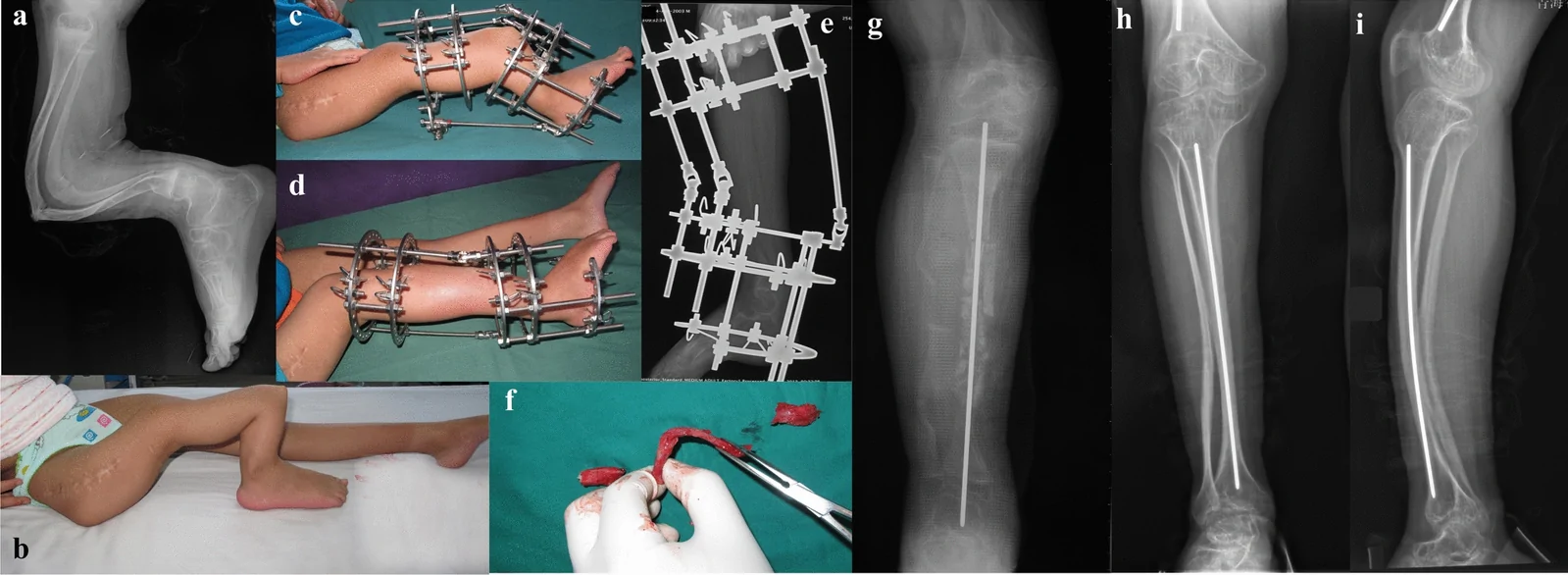
Surgical management of an osteogenesis imperfecta patient. **a** A 10-year-old boy with recurrent fractures and severe tibial bowing deformity accompanied by shortening; **b** marked limb shortening compared to the contralateral side; **c** stage I surgery involving the application of an Ilizarov external fixator for gradual distraction; **d** correction of the lower leg deformity; **e** preoperative radiograph revealing severe osteoporosis of the tibia and fibula; **f** intraoperative finding of a pliable tibia; **g** surgical procedure involving intramedullary fixation; **h**, **i** follow-up at 6 years postoperatively demonstrating normal tibial growth and development. The patient achieved the ability to stand and walk

The primary surgical treatment for long bones in OI is intramedullary rodding. Plates and screws alone are discouraged as they create stress risers. A modern technique combines unicortical locking plates with rods for rotational stability, with plates removed after healing. Rod design has evolved from static rods (e.g., Sofield, Rush), which require frequent revision, to telescopic models (Fig. 6). The Fassier-Duval (FD) telescopic rod is now the gold standard, offering a minimally invasive approach that reduces complications. Newer corkscrew-tipped and titanium rods are under investigation [139].

FD rods are associated with superior outcomes, with an 88% 4-year survival rate versus 40% for non-telescopic rods. They improve ambulation, motor function, and reduce fractures, especially when combined with bisphosphonates and rehabilitation. Upper extremity rodding enhances self-care. Surgical outcomes depend on OI severity. Type III OI often requires more revisions. The decision to rod is based on deformity, fracture frequency, and function; this requires multidisciplinary evaluation [140].

There are several limitations, including rod migration, bending, failure to telescope, and problematic distal fixation. Oversized rods can cause stress shielding. Experience from our center indicates that intramedullary nailing can potentially affect proximal femoral growth, leading to coxa valga, which warrants attention.

Future directions focus on improved distal fixation (e.g., cement and corkscrew designs), material innovation (e.g., titanium rods), combined techniques for complex cases, and personalized surgical timing. Based on our observations, future implant designs should also consider physeal impact.

In summary, surgical management of OI has been transformed by telescopic rodding (particularly FD rods) and a multidisciplinary approach. While significant improvements in function and reduced revisions are achieved, challenges in fixation, cost, and technique remain.

### Variability in response to bisphosphonate treatment

**Fig. 7:**
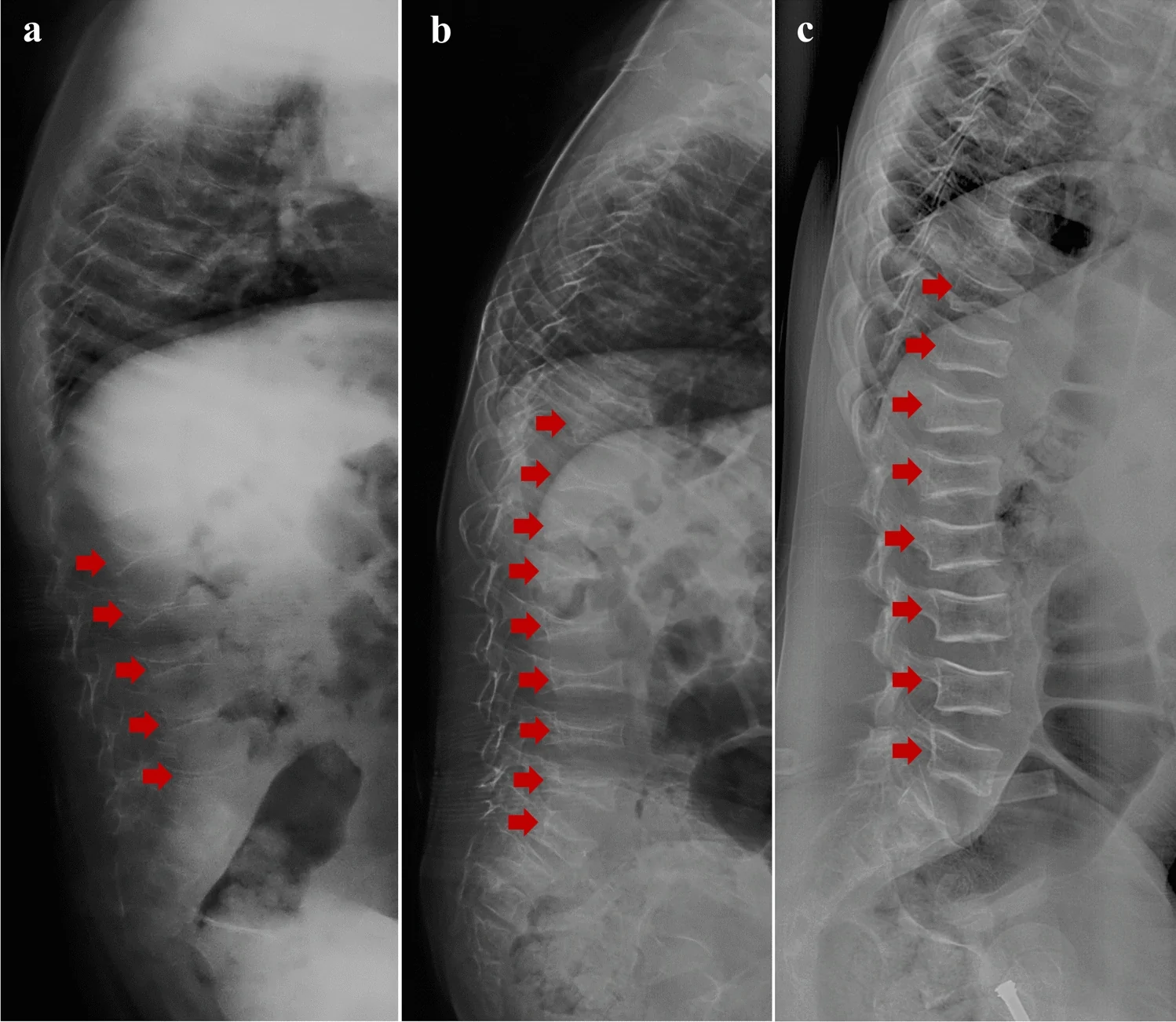
Radiographic changes in the spine of a patient with osteogenesis imperfecta type IV following bisphosphonate therapy. **a** Lateral spinal radiograph at 5 years of age, prior to bisphosphonate initiation. The image reveals severely low bone mineral density, with marked flattening and biconcave deformities of the vertebral bodies; **b** follow-up lateral radiograph after 1 year of bisphosphonate treatment. Partial restoration of vertebral body height and early remodeling are observed; **c** lateral radiograph after 5 years of continuous therapy. Significant recovery of vertebral body height and architecture is evident, demonstrating the positive long-term structural response to bisphosphonate treatment

Bisphosphonate therapy is a cornerstone of medical management in children with OI, demonstrating significant efficacy in improving BMD with a generally acceptable safety profile. The seminal work of Glorieux et al. in 1998 first established the benefits of cyclical intravenous pamidronate in severe OI [141]. There were dramatic increases in lumbar spine BMD and a substantial reduction in fracture rates [141]. However, the fracture reduction response across different bisphosphonates is not uniform. Whilst intravenous formulations (e.g., pamidronate, neridronate) consistently show a significant reduction in fracture risk, the evidence for oral bisphosphonates is weaker [142]. For instance, the large randomized controlled trial by Ward et al. in 2011 on alendronate demonstrated a significant increase in lumbar spine BMD but did not show a statistically significant reduction in the incidence of long-bone fracture compared to placebo [143]. Importantly, the therapeutic response also varies with OI genotype. A recent retrospective study by Charpié et al. in 2024 on non-*COL1* OI forms found that patients with OI type V responded uniformly to bisphosphonates with a significant decrease in fracture rate [144]. In contrast, responses were heterogeneous in patients with OI type VI, with some showing a paradoxical increase in fractures despite improved BMD. This highlights the critical role of underlying bone matrix quality beyond mere bone density. The efficacy of bisphosphonates in OI type XV is also limited (Fig. 7).

Regarding safety, the most common adverse effects are acute-phase reactions (fever, myalgia) following the first intravenous infusion and gastrointestinal discomfort with oral formulations; these are typically transient and manageable. Long-term safety data from extended cohort studies have raised no major concerns, though specific considerations like the risk of atypical femoral fractures or delayed bone healing are currently monitored.

### Emerging pharmacological therapies

#### Anti-RANKL monoclonal antibody: denosumab

Denosumab, a monoclonal antibody targeting RANKL, is approved for osteoporosis and inhibits osteoclast activity to increase BMD [145]. However, its efficacy and safety in patients with OI are not well-established [146].

Small pediatric OI studies show denosumab improves BMD and longitudinal growth [147, 148]. A significant concern is the rebound effect after discontinuation, characterized by increased bone turnover and disrupted mineral homeostasis, particularly hypercalcemia in children [149, 150]. A case series of eight OI patients found improved BMD without hypercalcemia, while another study in children noted only modest BMD increases without significant clinical improvement, alongside mild, transient alterations in mineral metabolism [151, 152].

Two recent studies compared denosumab to zoledronic acid (ZA). In adults with OI, denosumab improved BMD with comparable efficacy to ZA and a more favorable safety profile [153]. In contrast, a pediatric study revealed significant safety concerns [154]. While denosumab increased BMD and improved spinal morphometry, 31% of treated children developed rebound hypercalcemia, with nearly half of those cases progressing to hypercalcemic crisis. This rebound occurred approximately 4.7 months post-treatment, was more likely in children under five, and was associated with markedly elevated markers of bone turnover.

In conclusion, while denosumab has therapeutic potential in OI, its risk–benefit profile differs markedly between adults and children. The pronounced risk of rebound hypercalcemia in young children necessitates cautious use, stringent monitoring, and further research to optimize dosing and management strategies.

#### Anti-sclerostin monoclonal antibody: romosozumab

Romosozumab, a monoclonal antibody targeting sclerostin, promotes bone formation by inhibiting the Wnt pathway [56]. While approved for postmenopausal osteoporosis [155], its application in OI remains investigational, with efficacy and safety in this population yet to be established. Documented clinical experience is currently limited to two adult case reports. In a 64-year-old male with severe OI, romosozumab therapy for 12 months resulted in dramatic increases in BMD at the lumbar spine (22%) and total hip (136.4%) [156]. Similarly, a 52-year-old woman with mild OI showed substantial BMD improvements at the spine (10.3%) and hip (5.4%) after a 12-month course [157].

To evaluate its potential more systematically, a phase I trial (NCT04545554) has been completed in 25 pediatric OI patients, assessing pharmacokinetics and safety, though results are pending. An ongoing phase III trial (NCT05972551), expected to conclude in 2027, aims to directly compare romosozumab with bisphosphonates in 106 pediatric patients. This study will provide critical evidence regarding the comparative efficacy and safety in the OI population.

#### Anti-sclerostin monoclonal antibody: setrusumab

Setrusumab is an investigational fully human anti-sclerostin IgG2λ monoclonal antibody for the treatment of OI. In a 12-month, phase 2b, dose-finding trial (NCT03118570) in adults with type I/III/IV OI, no significant differences were found among the different dose groups in annualized fracture rates or radial trabecular volumetric BMD [158]. However, the 20 mg/kg group showed significantly increased bone failure load, and the 8 and 20 mg/kg groups had markedly improved bone stiffness [158]. Two ongoing trials (NCT05768854, NCT06636071) are evaluating the antifracture efficacy of setrusumab in pediatric OI, with NCT05768854 comparing setrusumab to intravenous bisphosphonates. NCT05312697, a long-term extension of NCT03118570, was terminated by the sponsor for non-safety-related reasons.

#### Anti-TGF-β monoclonal antibody: fresolimumab

Preclinical evidence identifies elevated TGF-β signaling as a key pathological mechanism in OI where it disrupts bone remodeling [159]. Indeed, inhibition of TGF-β has emerged as a promising therapeutic strategy. This is demonstrated by the anti-TGF-β antibody 1D11, which improves bone mass and strength in OI mouse models.

Translating this to the clinic, a phase I trial evaluated fresolimumab, a pan-TGF-β antibody, in adults with OI [57]. This antibody produced a biphasic effect: 1 mg/kg increased bone turnover, while 4 mg/kg was suppressive. Effects on lumbar spine BMD were variable, with some participants achieving increases of over 7%–8%, though responses were not uniform across the cohort.

Fresolimumab demonstrated a generally favorable safety profile in this OI population. No treatment-related serious adverse events occurred. The lower 1 mg/kg dose was associated with only mild, transient side effects like nausea and epistaxis. The higher 4 mg/kg dose resulted in more frequent but generally manageable adverse events, including headache, malaise, and minor bleeding episodes. These initial findings support further investigation of TGF-β inhibition for OI, though optimal dosing and long-term effects require careful evaluation.

### Cell-based therapies in osteogenesis imperfecta

Traditional OI treatments, such as bisphosphonates, are primarily palliative and do not address the underlying genetic defect. Cell therapy, particularly using mesenchymal stem cells (MSCs), has emerged as a strategy to directly target the pathological mechanisms of OI. Early attempts involved bone marrow transplantation, but the low survival rate of donor osteoblasts (1%***–***2%) and the complex marrow composition limited its efficacy [160]. Subsequent research shifted towards purified MSCs, which can differentiate into osteoblasts and modulate the bone microenvironment through paracrine signaling. MSCs from various sources, including bone marrow-derived MSCs (BMSCs), umbilical cord blood-derived MSCs, and fetal liver-derived MSCs (FL-MSCs), have been investigated [161–163]. FL-MSCs, in particular, demonstrate superior proliferative and osteogenic potential.

Clinical studies have shown promise. In one trial, children with severe OI who received BMSC infusions following a bone marrow transplant experienced a significant increase in growth rate (60%–94%), suggesting the engrafted cells contributed to bone formation. The TERCELOI trial demonstrated that repeated infusions of human leukocyte antigen (HLA)-haploidentical MSCs could reduce fracture incidence and improve quality of life. A landmark case of in utero transplantation of FL-MSCs in a severe OI fetus resulted in 7.4% engraftment, normalized bone morphology, and a postnatal period with minimal fractures [164]. However, the therapeutic effect waned as donor cells were depleted, leading to increased fracture rates after 1–2 years, highlighting the challenge of transient engraftment. Ongoing clinical trials such as BOOSTB4 and BOOST2B are further evaluating the safety and efficacy of prenatal and postnatal FL-MSC transplantation. Preliminary data indicate potential benefits for growth and bone density [165].

Despite this promise, significant challenges remain. These include the low engraftment rate of transplanted cells, the variable and often impaired osteogenic potential of patient-derived MSCs, the lack of standardized protocols for different MSC sources, and the high technical requirements for cell processing. While cell therapy offers new hope, it remains investigational, requiring breakthroughs in achieving durable engraftment and consistent therapeutic effects before becoming a mainstream treatment.

### Precision gene editing technologies

Current treatments for OI primarily manage symptoms but cannot cure the underlying genetic cause. Cell and gene therapies, particularly gene editing utilizing CRISPR-Cas9 technology, represent promising and potentially curative approaches.

Gene therapy for OI includes several approaches. While gene addition has limited utility due to the large size of the *COL1A1* and *COL1A2* genes, gene silencing offers a strategy to convert severe structural mutations into milder forms. Ultimately, precise gene editing to directly correct the causative mutations is the goal.

Substantial progress has been made using patient-derived induced pluripotent stem cells (iPSCs). Studies have successfully corrected various *COL1A1* and *COL1A2* mutations in iPSCs using CRISPR-Cas9. For instance, Cao et al. repaired a cis-double-variant in the *COL1A1* gene, which resulted in MSCs and osteoblasts derived from the corrected iPSCs exhibiting significantly improved osteogenic capacity [166]. This included normalized collagen expression, alkaline phosphatase activity, and mineralization. WGS confirmed the high accuracy of this strategy with no significant off-target effects [166]. Similarly, Jung et al. in 2021 demonstrated that correcting a *COL1A1* mutation (c.2523delT) via homology-directed repair (HDR) restored normal collagen protein levels and calcium deposition during osteogenic differentiation, bringing the function of the edited cells to a level comparable to healthy controls [167].

There is significant progress in the development of in vivo models. Indeed, Yang et al. developed an innovative therapy using recombinant adeno-associated virus serotype 9 (rAAV9) to deliver CRISPR-Cas9 components systemically to an OI mouse model [168]. Their strategy combined Cas9 with a "GeneRide" donor vector containing a corrective sequence, leveraging the HDR pathway for precise correction of a *COL1A2* mutation. This approach achieved a 12% HDR-based correction in bone tissue. Treated mice exhibited profound therapeutic benefits, including significantly improved bone mass, reduced skeletal deformities, enhanced fracture healing, and increased grip strength. Micro-CT analysis confirmed increases in trabecular bone volume and cortical thickness.

Despite this remarkable potential, several hurdles remain. These include enhancing the efficiency of gene editing in bone tissue, minimizing off-target effects for long-term safety, and ensuring the durability of the therapeutic effect. Future work should focus on optimizing delivery vectors, developing next-generation editors with improved precision, and conducting rigorous clinical trials to translate these promising strategies into safe and effective treatments for OI patients.

## Current challenges and future perspectives

OI is a genetically heterogeneous group of disorders characterized by bone fragility and a broad spectrum of connective tissue presentations. Historically classified based on clinical phenotypes, the advent of high-throughput sequencing has revolutionized the diagnosis of OI, revealing over 21 implicated genes and expanding the nosology to include up to 23 distinct types. This review systematically examines the genetic basis and molecular mechanisms underlying OI, highlighting mutations in collagen synthesis, post-translational modification, processing, mineralization, and osteoblast differentiation pathways. We discuss the critical shift from phenotypic to molecular etiologic classification, emphasizing how genetic insights refine prognosis, guide targeted therapies, such as bisphosphonates, anti-TGF-β agents, and sclerostin inhibitors, and inform genetic counseling. Emerging therapeutic strategies, including gene editing and cell therapies, offer promising avenues for correcting underlying genetic defects. The integration of molecular diagnostics and genotype–phenotype correlations is essential for advancing precision medicine in OI.

## Supplementary Information

Below is the link to the electronic supplementary material.

Supplementary file1 (DOCX 146 KB)

## Data Availability

Data sharing not applicable to this article as no datasets were generated or analyzed during the current study.
