# Supplementary material for: Pathogenesis and precision management of osteogenesis imperfecta: from genetic mechanisms to novel therapies
Source: World J Pediatr. 2026 Sep 30;22(9):982–1011. doi: 10.1007/s12519-026-01086-7 (PMC13653858; doi:10.1007/s12519-026-01086-7)
Supplement: Supplementary file 1 [file 12519_2026_1086_MOESM1_ESM.docx]

**Supplementary Table 1** Genetic classification and underlying pathogenesis of osteogenesis imperfecta-like disorders.

| **OI-like disorders** | **Genes** | **MIM** | **Molecular basis elucidated (year)** | **Chromosomal**  **location** | **Inheritance pattern** | **Pathogenesis** | **Animal model** |
| --- | --- | --- | --- | --- | --- | --- | --- |
| Cole-Carpenter syndrome type 1 | *P4HB* | 176790 | Rauch et al., 2015 [1] | 17q25.3 | AD | Procollagen processing and crosslinking | *P4hb^C402R/+^* mouse model [2] |
| Cole-Carpenter syndrome type 2 | *SEC24D* | 607186 | Garbes et al., 2015 [3] | 4q26 | AR | Osteoblast differentiation and function | *Sec24d^gt/gt^* Tg+ mice, early embryonic lethality [4] |
| Bruck syndrome type 2 | *PLOD2* | 601865 | van der Slot et al., 2003 [5] | 3q24 | AR | Procollagen processing and crosslinking | *Plod2^fs/fs^* mouse model [6] |
| Childhood/Juvenile-onset osteoporosis with developmental delay | *COPB2* | 606990 | Marom et al., 2021 [7] | 3q23 | AD | Procollagen processing and crosslinking | *Copb2^+/−^* mouse model [7] |
| Osteoporosis-pseudoglioma syndrome | *LRP5* | 603506 | Gong et al., 2001 [8] | 11q13.2 | AR | Wnt/β-catenin signaling pathway | *Lrp5^-/-^* mouse model [9, 10] |
| Gnathodiaphyseal dysplasia | *ANO5* | 608662 | Tsutsumi et al., 2004 [11] | 11p14.3 | AD | Osteoblast differentiation and function | *Ano5^-/-^* mouse model [12, 13]  *Ano5^KI/KI^* mouse model [14] |
| Bone mineral density quantitative trait locus 18 | *PLS3* | 300131 | van Dijk et al., 2013 [15] | Xq23 | XLD | Regulate cytoskeletal organization in a calcium-dependent manner | *Pls3^-/0^* mouse model [16]  *Pls3* knockdown zebrafish model [15] |
| Calvarial doughnut lesions with bone fragility | *SGMS2* | 611574 | Pekkinen et al., 2019 [17] | 4q25 | AD | Sphingomyelin metabolism | No model |
| Spondyloocular syndrome | *XYLT2* | 608125 | Munns et al., 2015 [18] | 17q21.33 | AR | Proteoglycan biosynthesis | No model |
| Geroderma osteodysplasticum | *GORAB* | 607983 | Hennies et al., 2008 [19] | 1q24.2 | AR | Extracellular matrix organization | Tamoxifen-inducible conditional *Gorab* knockout mouse model [20] |
| Autosomal recessive cutis laxa type IIB and IIIB | *PYCR1* | 179035 | Guernsey et al., 2009 [21]  Reversade et al., 2009 [22] | 17q25.3 | AR | Mitochondrial proline biosynthesis | No model |
| Autosomal recessive cutis laxa type IIA | *ATP6V0A2* | 611716 | Kornak et al., 2008 [23] | 12q24 | AR | Golgi acidification and function | No model |
| Wiedemann-Rautenstrauch syndrome | *POLR3A* | 614258 | Jay et al., 2016 [24] | 10q22.3 | AR | RNA polymerase III function | No model |
| Singleton-Merten syndrome 1 | *IFIH1* | 606951 | Rutsch et al., 2015 [25] | 2q24.2 | AD | Innate immune signaling | *Ifih1^gs/+^* mouse model [26] |

*OI* osteogenesis imperfecta, *MIM* Mendelian Inheritance in Man number, *AD* autosomal dominant, *AR* autosomal recessive, *XLD* X-linked dominant, *P4HB p*rocollagen-proline, 2-oxoglutarate-4-dioxygenase, beta subunit, *SEC24D* sec24-related fene family, member D, *PLOD2* procollagen-lysine, 2-oxoglutarate 5-dioxygenase 2, *COPB2* coatomer protein complex, subunit beta-2, *LRP5* low density lipoprotein receptor-related protein 5, *ANO5 a*noctamin 5, *PLS3* plastin 3, *SGMS2* sphingomyelin synthase 2, *XYLT2* xylosyltransferase 2, *GORAB* golgin, rab6-interacting, *PYCR1* pyrroline-5-carboxylate reductase 1, *ATP6V0A2* ATPase, H+ transporting, lysosomal, V0 subunit A2, *POLR3A p*olymerase III, RNA, subunit A, *IFIH1* interferon-induced helicase C domain-containing protein 1, *gt* gene trap, *Tg* transgenic, *KI* knock-in.

**Supplementary Table 2** Distinct features and clinical severity of osteogenesis imperfecta-like disorders.

| **OI-like disorders** | **MIM** | **First description** | **Genes** | **Severity of skeletal involvement** | **Distinctive features of bone abnormalities** | **Extraskeletal manifestations** |
| --- | --- | --- | --- | --- | --- | --- |
| Cole-Carpenter syndrome type 1 | 112240 | Cole et al., 1987 [27] | *P4HB* | Severe | Bone fragility, craniosynostosis, short stature, severe scoliosis | Ocular proptosis, hydrocephalus |
| Cole-Carpenter syndrome type 2 | 616294 | Garbes et al., 2015 [3] | *SEC24D* | Variable severity | Recurrent fractures, osteopenia, long bone deformities, craniosynostosis | Ocular proptosis, hydrocephalus, midface hypoplasia, micrognathia |
| Bruck syndrome type 2 | 609220 | van der Slot et al., 2003 [5] | *PLOD2* | Mild to severe | Increased bone fragility, congenital joint contractures, short stature | Inguinal hernia in males, pterygia |
| Childhood/Juvenile-Onset Osteoporosis with Developmental Delay | 619884 | Marom et al., 2021 [7] | *COPB2* | Moderate | Osteopenia/osteoporosis, recurrent fractures | Microcephaly, neurodevelopmental impairment |
| Osteoporosis-pseudoglioma syndrome | 259770 | Gong et al., 2001 [8] | *LRP5* | Moderate to severe | Recurrent fractures, vertebral compression, kyphoscoliosis, short stature | Pseudoglioma and retinal defects |
| Gnathodiaphyseal dysplasia | 166260 | Tsutsumi et al., 2004 [11] | *ANO5* | Moderate | Bone fragility, frequent fractures, diaphyseal sclerosis/cortical thickening of long bones | Purulent osteomyelitis, cementoossifying fibroma of the jawbones |
| Bone mineral density quantitative trait locus 18 | 300910 | van Dijk et al., 2013 [15] | *PLS3* | Asymptomatic to moderate | Low BMD, recurrent long-bone fractures, vertebral compression fractures | None |
| Calvarial doughnut lesions with bone fragility, with or without spondylometaphyseal dysplasia | 126550 | Pekkinen et al., 2019 [17] | *SGMS2* | Mild to severe | Low BMD, multiple peripheral and spinal fractures, sclerotic "doughnut-shaped" cranial lesions | Peripheral facial nerve palsies |
| Spondyloocular syndrome | 605822 | Munns et al., 2015 [18] | *XYLT2* | Moderate to severe | Bone fragility | Ocular abnormalities, hearing impairment, cardiac defects |
| Geroderma osteodysplasticum | 231070 | Hennies et al., 2008 [19] | *GORAB* | Moderate to severe | Osteopenia, bowed long bones, vertebral compression fractures | Wrinkled skin (particularly on hands, feet, and abdomen) |
| Autosomal recessive cutis laxa type IIB and IIIB | 612940 614438 | Guernsey et al., 2009 [21]  Reversade et al., 2009 [22] | *PYCR1* | Variable severity | Osteopenia | Wrinkled skin, joint laxity,intrauterine growth restriction, neurodevelopmental abnormalities |
| Autosomal recessive cutis laxa type IIA | 219200 | Kornak et al., 2008 [23] | *ATP6V0A2* | Variable severity | Skeletal abnormalities, cobblestone-like brain malformations | Loose, inelastic skin, craniofacial dysmorphism, developmental delay, intellectual disability, seizures |
| Wiedemann-Rautenstrauch syndrome | 264090 | Jay et al., 2016 [24] | *POLR3A* | Mild to severe | Craniofacial abnormalities (pseudohydrocephaly, wide fontanels, sparse hair), dental anomalies | Intrauterine growth retardation, lipodystrophy with abnormal fat distribution, ataxia and tremor |
| Singleton-Merten syndrome 1 | 182250 | Rutsch et al., 2015 [25] | *IFIH1* | Moderate to severe | Root hypoplasia and premature tooth loss, osteoporosis and acroosteolysis | Vascular calcifications (aortic valve and ascending aorta) |

*BMD* bone mineral density, *OI* osteogenesis imperfecta, *MIM* Mendelian Inheritance in Man number, *AD* autosomal dominant, *AR* autosomal recessive, *XLD* X-linked dominant, *P4HB p*rocollagen-proline, 2-oxoglutarate-4-dioxygenase, beta subunit, *SEC24D* sec24-related gene family, member D, *PLOD2 p*rocollagen-lysine, 2-oxoglutarate 5-dioxygenase 2, *COPB2 c*oatomer protein complex, subunit beta-2, *LRP5* low density lipoprotein receptor-related protein 5, *ANO5 a*noctamin 5, *PLS3 p*lastin 3, *SGMS2 s*phingomyelin synthase 2, *XYLT2* xylosyltransferase 2, *GORAB* golgin, rab6-interacting, *PYCR1 p*yrroline-5-carboxylate Reductase 1, *ATP6V0A2* ATPase, H+ transporting, lysosomal, V0 subunit A2, *POLR3A p*olymerase III, RNA, subunit A, *IFIH1* interferon-induced helicase C domain-containing protein 1.

**Reference**

[1] Rauch F, Fahiminiya S, Majewski J, Carrot-Zhang J, Boudko S, Glorieux F, et al., Cole-Carpenter syndrome is caused by a heterozygous missense mutation in P4HB. Am J Hum Genet. 2015;96:425-31. doi:10.1016/j.ajhg.2014.12.027.
[2] Xu S, Xu Y, Wang Z, Wei Z, Mei Y, Cao Y, et al., Endoplasmic reticulum stress causes long bone shortening in P4hb(C402R/+) mice: A mouse model exhibiting significant features of cole-carpenter syndrome driven by P4HB mutations. Biochim Biophys Acta Mol Basis Dis. 2025;1871:167663. doi:10.1016/j.bbadis.2025.167663.
[3] Garbes L, Kim K, Rieß A, Hoyer-Kuhn H, Beleggia F, Bevot A, et al., Mutations in SEC24D, encoding a component of the COPII machinery, cause a syndromic form of osteogenesis imperfecta. Am J Hum Genet. 2015;96:432-9. doi:10.1016/j.ajhg.2015.01.002.
[4] Baines AC, Adams EJ, Zhang B, Ginsburg D. Disruption of the Sec24d gene results in early embryonic lethality in the mouse. PLoS One. 2013;8:e61114. doi:10.1371/journal.pone.0061114.
[5] van der Slot AJ, Zuurmond AM, Bardoel AF, Wijmenga C, Pruijs HE, Sillence DO, et al., Identification of PLOD2 as telopeptide lysyl hydroxylase, an important enzyme in fibrosis. J Biol Chem. 2003;278:40967-72. doi:10.1074/jbc.M307380200.
[6] Kot A, Chun C, Martin JH, Wachtell D, Hudson D, Weis M, et al., Loss of the long form of Plod2 phenocopies contractures of Bruck syndrome-osteogenesis imperfecta. J Bone Miner Res. 2024;39:1240-52. doi:10.1093/jbmr/zjae124.
[7] Marom R, Burrage LC, Venditti R, Clément A, Blanco-Sánchez B, Jain M, et al., COPB2 loss of function causes a coatopathy with osteoporosis and developmental delay. Am J Hum Genet. 2021;108:1710-24. doi:10.1016/j.ajhg.2021.08.002.
[8] Gong Y, Slee RB, Fukai N, Rawadi G, Roman-Roman S, Reginato AM, et al., LDL receptor-related protein 5 (LRP5) affects bone accrual and eye development. Cell. 2001;107:513-23. doi:10.1016/s0092-8674(01)00571-2.
[9] Chang MK, Kramer I, Keller H, Gooi JH, Collett C, Jenkins D, et al., Reversing LRP5-dependent osteoporosis and SOST deficiency-induced sclerosing bone disorders by altering WNT signaling activity. J Bone Miner Res. 2014;29:29-42. doi:10.1002/jbmr.2059.
[10] Kedlaya R, Veera S, Horan DJ, Moss RE, Ayturk UM, Jacobsen CM, et al., Sclerostin inhibition reverses skeletal fragility in an Lrp5-deficient mouse model of OPPG syndrome. Sci Transl Med. 2013;5:211ra158. doi:10.1126/scitranslmed.3006627.
[11] Tsutsumi S, Kamata N, Vokes TJ, Maruoka Y, Nakakuki K, Enomoto S, et al., The novel gene encoding a putative transmembrane protein is mutated in gnathodiaphyseal dysplasia (GDD). Am J Hum Genet. 2004;74:1255-61. doi:10.1086/421527.
[12] Liu X, Wang S, Liang C, Zhang S, Liu S, Hu Y. Ano5 deficiency disturbed bone formation by inducing osteoclast apoptosis in Gnathodiaphyseal dysplasia. Exp Cell Res. 2025;447:114493. doi:10.1016/j.yexcr.2025.114493.
[13] Wang X, Liu X, Dong R, Liang C, Reichenberger EJ, Hu Y. Genetic Disruption of Anoctamin 5 in Mice Replicates Human Gnathodiaphyseal Dysplasia (GDD). Calcif Tissue Int. 2019;104:679-89. doi:10.1007/s00223-019-00528-x.
[14] Li H, Wang X, Chen E, Liu X, Ma X, Miao C, et al., Introduction of a Cys360Tyr Mutation in ANO5 Creates a Mouse Model for Gnathodiaphyseal Dysplasia. J Bone Miner Res. 2022;37:515-30. doi:10.1002/jbmr.4481.
[15] van Dijk FS, Zillikens MC, Micha D, Riessland M, Marcelis CL, de Die-Smulders CE, et al., PLS3 mutations in X-linked osteoporosis with fractures. N Engl J Med. 2013;369:1529-36. doi:10.1056/NEJMoa1308223.
[16] Yorgan TA, Sari H, Rolvien T, Windhorst S, Failla AV, Kornak U, et al., Mice lacking plastin-3 display a specific defect of cortical bone acquisition. Bone. 2020;130:115062. doi:10.1016/j.bone.2019.115062.
[17] Pekkinen M, Terhal PA, Botto LD, Henning P, Mäkitie RE, Roschger P, et al., Osteoporosis and skeletal dysplasia caused by pathogenic variants in SGMS2. JCI Insight. 2019;4. doi:10.1172/jci.insight.126180.
[18] Munns CF, Fahiminiya S, Poudel N, Munteanu MC, Majewski J, Sillence DO, et al., Homozygosity for frameshift mutations in XYLT2 result in a spondylo-ocular syndrome with bone fragility, cataracts, and hearing defects. Am J Hum Genet. 2015;96:971-8. doi:10.1016/j.ajhg.2015.04.017.
[19] Hennies HC, Kornak U, Zhang H, Egerer J, Zhang X, Seifert W, et al., Gerodermia osteodysplastica is caused by mutations in SCYL1BP1, a Rab-6 interacting golgin. Nat Genet. 2008;40:1410-2. doi:10.1038/ng.252.
[20] Chan WL, Bucher CH, Goldes J, Ma AC, Steiner M, Willie BM, et al., Targeting TGF-β signaling, oxidative stress, and cellular senescence rescues osteoporosis in gerodermia osteodysplastica. Aging Cell. 2024;23:e14322. doi:10.1111/acel.14322.
[21] Guernsey DL, Jiang H, Evans SC, Ferguson M, Matsuoka M, Nightingale M, et al., Mutation in pyrroline-5-carboxylate reductase 1 gene in families with cutis laxa type 2. Am J Hum Genet. 2009;85:120-9. doi:10.1016/j.ajhg.2009.06.008.
[22] Reversade B, Escande-Beillard N, Dimopoulou A, Fischer B, Chng SC, Li Y, et al., Mutations in PYCR1 cause cutis laxa with progeroid features. Nat Genet. 2009;41:1016-21. doi:10.1038/ng.413.
[23] Kornak U, Reynders E, Dimopoulou A, van Reeuwijk J, Fischer B, Rajab A, et al., Impaired glycosylation and cutis laxa caused by mutations in the vesicular H+-ATPase subunit ATP6V0A2. Nat Genet. 2008;40:32-4. doi:10.1038/ng.2007.45.
[24] Jay AM, Conway RL, Thiffault I, Saunders C, Farrow E, Adams J, et al., Neonatal progeriod syndrome associated with biallelic truncating variants in POLR3A. Am J Med Genet A. 2016;170:3343-46. doi:10.1002/ajmg.a.37960.
[25] Rutsch F, MacDougall M, Lu C, Buers I, Mamaeva O, Nitschke Y, et al., A specific IFIH1 gain-of-function mutation causes Singleton-Merten syndrome. Am J Hum Genet. 2015;96:275-82. doi:10.1016/j.ajhg.2014.12.014.
[26] Soda N, Sakai N, Kato H, Takami M, Fujita T. Singleton-Merten Syndrome-like Skeletal Abnormalities in Mice with Constitutively Activated MDA5. J Immunol. 2019;203:1356-68. doi:10.4049/jimmunol.1900354.
[27] Cole DE, Carpenter TO. Bone fragility, craniosynostosis, ocular proptosis, hydrocephalus, and distinctive facial features: a newly recognized type of osteogenesis imperfecta. J Pediatr. 1987;110:76-80. doi:10.1016/s0022-3476(87)80292-5.
